# ﻿Novel discoveries of Xylariomycetidae (Ascomycota) taxa from peat swamp forests and other terrestrial habitats in Thailand

**DOI:** 10.3897/mycokeys.107.127749

**Published:** 2024-08-07

**Authors:** Omid Karimi, Naghmeh Afshari, Raheleh Asghari, Qirui Li, K. W. Thilini Chethana, Kevin D. Hyde, Fatimah O. Alotibi

**Affiliations:** 1 School of Science, Mae Fah Luang University, Chiang Rai 57100, Thailand; 2 Center of Excellence in Fungal Research, Mae Fah Luang University, Chiang Rai 57100, Thailand; 3 Department of Biology, Faculty of Science, Chiang Mai University, Chiang Mai 50200, Thailand; 4 State Key Laboratory of Functions and Applications of Medicinal Plants, Guizhou Medical University, Guiyang 550004, China; 5 The High Efficacy Application of Natural Medicinal Resources Engineering Center of Guizhou Province (The Key Laboratory of Optimal Utilization of Natural Medicine Resources), School of Pharmaceutical Sciences, Guizhou Medical University, Guiyang, China; 6 Department of Botany and Microbiology, College of Science, King Saud University, P.O. Box 22452, Riyadh 11495, Saudi Arabia

**Keywords:** Fungal diversity, multi-gene phylogeny, novel species, Sordariomycetes, taxonomy

## Abstract

In a comprehensive survey of fungi conducted in the northern (Chiang Rai Province) and southern (Narathiwat Province) regions of Thailand, several xylariales-like specimens were discovered. Through the integration of molecular phylogeny and morphological analyses, one previously undocumented taxon, *Oxydothisnarathiwatensis***sp. nov.**, was identified, along with *Xylariabawanglingensis* and *Hypoxylonhypomiltum* as new host and geographical records from *Afzeliaxylocarpa*, and *Dalbergiacana*, respectively. In addition, *Annulohypoxylonthailandicum* was identified as a new host record from *Swieteniamacrophylla* in Thailand. The morphological characters, including ascomata, asci, and ascospores, were compared with known *Oxydothis*, *Xylaria*, *Hypoxylon*, and *Annulohypoxylon* species. Multi-locus phylogenetic analyses based on ITS, LSU, and SSU (for Oxydothidaceae), ITS, *rpb2*, *tub2*, and *act* (for Xylariaceae), and ITS, LSU, *rpb2*, and *tub2* (for Hypoxylaceae) gene regions were carried out to refine the taxonomic classifications of these specimens further. This research contributes to understanding fungal diversity in these ecologically significant regions, highlighting insights into the relationships among xylariales-like species.

## ﻿Introduction

Xylariomycetidae, introduced by Eriksson and Winka (1997), is one of the largest subclasses in Ascomycota and belongs to the class Sordariomycetes (Hyde et al. 2020a). This subclass encompasses three orders and more than 35 families (Wijayawardene et al. 2022). Among these, Xylariaceae and Hypoxylaceae stand out as two particularly significant families (Wendt et al. 2018; Voglmayr et al. 2019; Hyde et al. 2020a, 2020b; Sun et al. 2021; Hernãndez-Restrepo et al. 2022; Sugita et al. 2022, 2024; Wijayawardene et al. 2022), while Oxydothidaceae is one of the poorly represented families in terms of sequence data, with fewer than 20 species having sequence data (Konta et al. 2016; Hyde et al. 2020b; Senanayake et al. 2023).

Xylariaceae stands out as one of the largest and most diverse families within the Xylariales, a fact highlighted by various studies (Ju and Rogers 1996; Fröhlich and Hyde 2000; Tang et al. 2009; Stadler et al. 2013; Koyani et al. 2016; Daranagama et al. 2018; Konta et al. 2020; Samarakoon et al. 2022; Li et al. 2024a, 2024b). *Xylaria* is the largest genus in Xylariaceae, with *Xylariahypoxylon* as the type species (Peršoh et al. 2009; Wijayawardene et al. 2022). The majority of xylariaceous species function as endophytes or saprobes, thriving on fallen woods, leaves, fruits, seeds, dung, soil, and termite nests. Notably, a few are recognized as plant pathogens (Rogers 2000; Okane et al. 2008; Stadler et al. 2013; Husbands et al. 2018; Pourmoghaddam et al. 2022).

The family Hypoxylaceae, initially proposed by de Candolle (cf. de Lamarck and de Candolle 1805), was later synonymized under Xylariaceae but resurrected and emended by Wendt et al. (2018). It encompasses approximately 422 species across 19 genera, notably *Hypoxylon* and *Annulohypoxylon* (Hyde et al. 2020a; Wijayawardene et al. 2022), thriving in diverse climates and primarily associated with dead dicotyledonous wood. *Hypoxylon*, the type genus introduced by Bulliard (1791), consists of 235 species, often found in warmer regions, particularly the neotropics (Ju and Rogers 1996; Daranagama et al. 2018). Traditionally, *Hypoxylon* was characterized by nodulisporium-like anamorphs and specific stromatal features (Ju and Rogers 1996), but modern understanding integrates morphology with molecular phylogeny and stromatal pigment profile (Hsieh et al. 2005; Kuhnert et al. 2015). As a result, Hypoxylonsect.Annulata sensu evolved into the genus *Annulohypoxylon* following phylogenetic analyses (Hsieh et al. 2005), with subsequent revisions and species descriptions (Kuhnert et al. 2017). *Annulohypoxylon*, predominantly characterized by ostioles encircled by an annulated disc, comprises 69 species (https://www.speciesfungorum.org), exhibiting diverse ecological roles (Pažoutová et al. 2013; Daranagama et al. 2018; Wendt et al. 2018; Cruz et al. 2020). Both *Hypoxylon* and *Annulohypoxylon* are known for harboring a diverse array of secondary metabolites, some of which exhibit promising agricultural and medicinal properties (Scherlach et al. 2010; Helaly et al. 2018; Becker and Stadler 2021).

Oxydothidaceae was introduced by Konta et al. (2016) to accommodate *Oxydothis* species. *Oxydothis* was introduced by Penzig and Saccardo (1897) with the type species *O.grisea* and two other species, *O.nigricans* and *O.maculosa*, placed in the family Amphisphaeriaceae (sensu Eriksson and Hawksworth 1991). Hyde (1993c) reviewed the genus and proposed that *Oxydothis* should be transferred from Amphisphaeriaceae to the Hyponectriaceae based on ascus, ascospore, and peridium morphologies. He also emphasized the consistency of ascus and ascospore morphology, which is essential for identifying species, and compared it with the closely related genera *Ceriospora*, *Frondispora*, *Lasiobertia* and *Leiosphaerella* (Hyde 1993c). Kang et al. (1999) transferred the genus to Clypeosphaeriaceae, but Jeewon et al. (2003) suggested that it was related to *Leiosphaerella* (Xylariales, genera incertae sedis) based on DNA sequence data. Konta et al. (2016) transferred *Oxydothis* to Oxydothidaceae (Xylariales). Besides confirming *Oxydothis* placement in Oxydothidaceae, the family was accepted in Amphisphaeriales by Samarakoon et al. (2022) and Wijayawardene et al. (2022). Currently, *Oxydothis* comprises 80 species listed in the Species Fungorum (https://www.speciesfungorum.org, accessed in June 2024). *Oxydothis* species are predominantly found on bamboo, palms, and *Pandanus*, primarily as saprobes (Hyde 1993a, 1993b, 1994a, 1994b; Wang and Hyde 1999; Fröhlich and Hyde 2000; Wong and Hyde 2001; Taylor and Hyde 2003; Shenoy et al. 2005; Hidayat et al. 2006; Tibpromma et al. 2018), with occasional occurrences as pathogens (Fröhlich and Hyde 1994) and endophytes (Hyde 1994b; Taylor et al. 1999).

This paper describes one new species, *Oxydothisnarathiwatensis*, and presents three new records, *Xylariabawanglingensis*, *Hypoxylonhypomiltum*, and *Annulohypoxylonthailandicum*. Additionally, molecular sequence data and phylogenetic information are provided for these taxa.

## ﻿Materials and methods

### ﻿Sampling and morphological studies

Xylariaceae and Hypoxylaceae specimens were collected in 2022 from dead wood of *Afzeliaxylocarpa*, *Swieteniamacrophylla*, and *Dalbergiacana* at Doi Tung National Park and Mae Fah Luang University, Chiang Rai, Thailand. Oxydothidaceae specimens were collected from submerged rachis of *Eleiodoxaconferta* from a peat swamp forest in Narathiwat Province, Thailand, in 2023. Morphological observations and single spore isolations followed the procedures outlined in Senanayake et al. (2020). The samples were cultured in different media, including potato dextrose agar (PDA), malt extract agar (MEA), and oatmeal agar (OA). Herbarium specimens were deposited in the Herbarium of Mae Fah Luang University (MFLU), Chiang Rai, Thailand. Living cultures are deposited in the Mae Fah Luang University Culture Collection (MFLUCC), Chiang Rai, Thailand. Faces of fungi and Index Fungorum numbers are registered as described in Jayasiri et al. (2015) and Index Fungorum (https://www.indexfungorum.org), respectively. Descriptions and collection details are added to the Greater Mekong Subregion database (Chaiwan et al. 2021).

### ﻿DNA extraction, PCR amplification, and Phylogenetic analyses

Fresh mycelia were used to extract genomic DNA using the Mega Genomic DNA Extraction Kit, following the manufacturer’s instructions (Omega Bio-tek Inc, The United States). The PCR amplifications were conducted for six loci, including Internal transcribed spacer (ITS), large subunit rDNA (LSU), nuclear small subunit rDNA (SSU), RNA polymerase II subunit (*rpb2*), beta-tubulin (tub2), and actin (*act*) (Table 1). Sequence data of related taxa (Xylariaceae, Hypoxylaceae, and Oxydothidaceae) were downloaded from the GenBank database (Suppl. material 1: tables S1–S3). The phylogenetic analyses based on maximum likelihood (ML) and Bayesian inferences were conducted for both the individual datasets and the concatenated dataset of six loci. The CIPRES Science Gateway platform (Miller et al. 2010) was used for performing the maximum likelihood (ML) phylogenetic analysis, utilizing the RAxMLHPC2 tool on the XSEDE (v. 8.2.10) platform (Stamatakis 2014). The analysis employed the GTRGAMMA substitution model and the rapid bootstrap analysis algorithm for 1000 replicates. The Bayesian analysis was performed using MrBayes v. 3.2.6 on XSEDE at the CIPRES Science Gateway, with the analysis set to two parallel runs, four Markov chains, and run for 10,000,000 generations. The pairwise homoplasy index (PHI) test was conducted using the combined sequence dataset comprising ITS, LSU, and SSU genes of closely related species using Split Tree version 4.18.2 (Huson and Bryant 2006) to evaluate the recombination level. The generated phylograms were visualized using FigTree v. 1.4.0 (Rambaut 2015), and annotations were added using Adobe Photoshop CS6 Extended version 10.0 software (Adobe Systems, United States).

**Table 1.** Primers and PCR conditions used for each gene region in the current study.

Gene region	Primers	PCR Condition	References
ITS	ITS5/ITS4	Initial denaturation: 94 °C (3 min); 35 cycles of denaturation: 95 °C (1 min), annealing: 53 °C (55 sec) and extension 72 °C (2 min); Final extension: 72 °C (10 min)	White et al. (1990)
LSU	LR0R/LR5	Initial denaturation: 94 °C (5 min); 35 cycles of denaturation: 94 °C (30 sec), annealing: 55 °C (50 sec) and extension 72 °C (2 min); Final extension: 72 °C (10 min)	Vilgalys and Hester (1990); Rehner and Samuels (1994)
SSU	NS1/NS4	Initial denaturation: 94 °C (3 min); 35 cycles of denaturation: 94 °C (30 sec), annealing: 55 °C (50 sec) and extension 72 °C (2 min); Final extension: 72 °C (10 min)	White et al. (1990)
*rpb*2	fRPB2-5F/fRPB2-7cR	Initial denaturation: 95 °C (5 min); 35 cycles of denaturation: 95 °C (1 min), annealing: 52°C (1 min) and extension 72 °C (2 min); Final extension: 72 °C (10 min)	Liu et al. (1999)
*tub2*	T1/T22	Initial denaturation: 95 °C (2 min); 35 cycles of denaturation: 95 °C (1 min), annealing: 54 °C (1.5 min) and extension 72 °C (2 min); Final extension: 72 °C (10 min)	O’Donnell et al. (1998); Hsieh et al. (2005)
*act*	ACT-512F/ACT-738R	Initial denaturation: 95 °C (5 min); 35 cycles of denaturation: 95 °C (1 min), annealing: 55 °C (30 sec) and extension 72 °C (1 min); Final extension: 72 °C (10 min)	Carbone and Kohn (1999)

## ﻿Results

### ﻿Phylogenetic analysis of Xylariaceae

**Figure 1.**
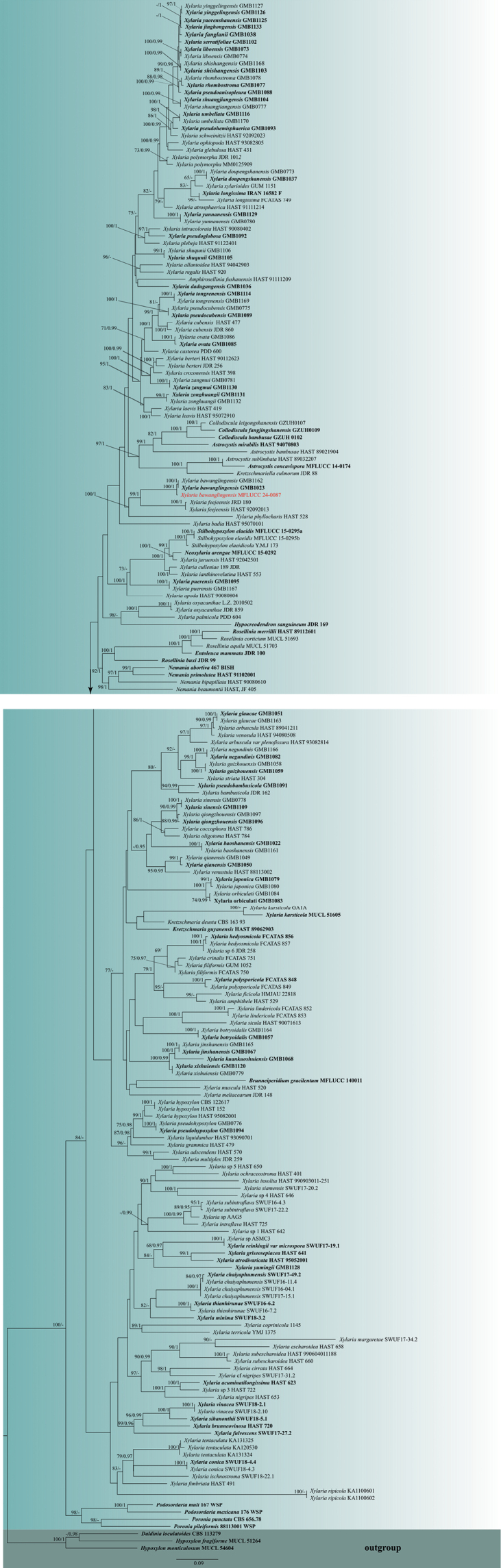
RAxML tree based on the analysis of a combined ITS, *rpb2*, *tub2*, and *act* dataset. ML bootstrap supports (MLBS) equal to or higher than 65% and Bayesian posterior probabilities (BYPP) equal to or greater than 0.95 are given near the nodes. Newly generated isolate of the current study is in red, and ex-types are in bold. The tree is rooted to *Daldinialoculatoides*, *Hypoxylonfragiforme* and *Hypoxylonmonticulosum*.

The combined ITS, *rpb2*, *tub2*, and *act* dataset consisted of 213 isolates belonging to 151 Xylariaceae taxa, with *Hypoxylonfragiforme* (MUCL 51264), *Hypoxylonmonticulosum* (MUCL 54604), and *Daldinialoculatoides* (CBS:113279) as outgroup taxa (Suppl. material 1: table S1). The final alignment comprised 3,726 characters (ITS: 606 bp, *rpb2*: 1,191 bp, *tub2*: 1,627 bp, *act*: 302 bp), including gaps. The final ML optimization likelihood value of the best RAxML tree (Fig. 1) was -136336.552252, and the matrix had 2,610 distinct alignment patterns, with 32.19% undetermined characters or gaps. Estimated base frequencies were as follows: A = 0.234039, C = 0.279027, G = 0.241491, T = 0.245443; substitution rates AC = 1.325774, AG = 5.176867, AT = 1.193683, CG = 1.131857, CT = 6.488876, GT = 1.000000; gamma distribution shape parameter α = 0.409124. The RAxML and Bayesian analyses yielded almost similar tree topologies. The topology of our phylogenetic tree is nearly identical to previous publications (Friebes et al. 2022; Pan et al. 2022; Ju et al. 2023; Karimi et al. 2023; Li et al. 2024b) with minor differences, which may be due to different taxon sampling, as well as different studies that have explored the taxonomy of *Xylaria* using various combinations of gene regions, such as ITS, *act*, *tub2*, and *rpb2* combination (Garcia-Aroca et al. 2021), *act* and *tub2* combination (Wangsawat et al. 2021), *tub2*, *act*, and *rpb2* combination (Hsieh et al. 2020, 2022) or ITS, *tub2*, and *rpb2* combination (Pan et al. 2022).

### ﻿Taxonomy

#### ﻿ Xylaria bawanglingensis

Taxon classificationFungiXylarialesXylariaceae

Y.P. Wu & Q.R. Li, J. Syst. Evol. 16 (2024)

BB7A5D51-C64B-553A-8F32-19AF68B550F0

Index Fungorum: IF849696

Facesoffungi Number: FoF16035

Figs 1 , 2

##### Description.

***Saprobic*** on dead wood of *Afzeliaxylocarpa* (Fabaceae). ***Sexual morph***: ***Stromata*** 0.5–2 × 0.1–0.3 cm (x– = 1.2 × 0.17 cm, n = 15), solitary or in a small group, not branched or rarely branched at the base, cylindrical to obclavate, narrowing towards the apex, and sometimes with a tomentum; surface rough, wrinkled, slightly cracked, gray to blackish and interior yellow. ***Perithecia*** 165–380 µm diam., (x– = 285 µm, n = 15), immersed, subglobose, ostioles inconspicuous. ***Paraphyses*** 5–10 µm width (x– = 8 µm, n = 20), cylindrical, septate. ***Asci*** 68–153 × 4–5.5 µm (x– = 109 × 4.8 µm, n = 30), 8-spored, cylindrical, and spore bearing part 67–87 µm long. Apical apparatus 0.2–2 × 1–2 µm (x– = 1.3 × 1.5 µm, n = 15), bluing in Melzer’s iodine reagent. ***Ascospores*** 8–12 × 2–5 µm (x– = 10 µm × 4 µm, n = 70), uniseriate, ellipsoid, subhyaline, light brown to dark brown, smooth, guttulate, germ slit apparent, sigmoid, almost full length of spore. ***Asexual morph***: Undetermined.

##### Culture characters.

Colonies grown on PDA, reaching 30 mm in diameter after 15 days at 25 °C, under dark conditions, mycelium superficial to immersed, without pigment diffusion, sporulation and concentric zones, white on the top and back views.

**Figure 2.**
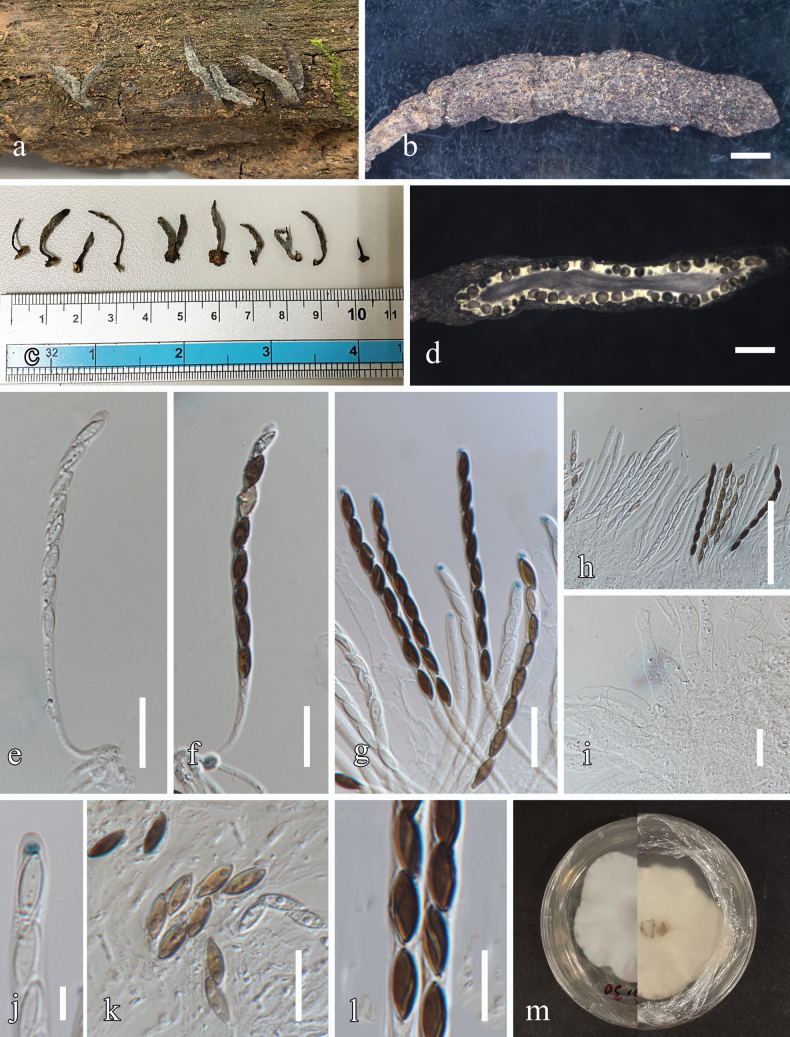
*Xylariabawanglingensis* (MFLU 24-0018, new host and geographical record) **a** stromata on host **b, c** stromata **d** longitudinal section through the stroma **e–h** asci **i** paraphyses **j** apical ring bluing in Melzer’s reagent **k, l** ascospores **m** upper view and reverse view of the one-week-old colony on PDA. Scale bars: 1 mm (**b, d**); 20 µm (**e–g**); 70 µm (**h**); 20 µm (**i**); 5 µm (**j**); 10 µm (**k, l**).

##### Material examined.

Thailand, Doi Tung National Park, Chiang Rai, on dead wood of *Afzeliaxylocarpa*, 27 September 2022, N. Afshari 5C3T2R2 (MFLU 24-0018), living culture MFLUCC 24-0087.

##### Known distribution.

China (Li et al. 2024b), and Thailand (This study).

##### Known hosts.

*Afzeliaxylocarpa* (This study).

##### Notes.

Our collection (MFLU 24-0018) morphologically resembles the *Xylariabawanglingensis* (GMB1023) in having solitary, fertile inflated, roughened surface and gray stromata with interior yellow, subglobose ostiolate perithecia, cylindrical asci with a J+ apical ring and unicellular ascospores with spore‐length sigmoid germ slit and almost similar sized stromata, perithecia, asci and ascospores. In the phylogenic analyses (Fig. 1), our collection (MFLUCC 24-0087) clustered with *Xylariabawanglingensis* (GMB1023, GMB1162) with 100% ML bootstrap support and 1.00 posterior probability support. In this study, we report our isolate (MFLU 24-0018) as a new host and geographical record of *Xylariabawanglingensis* on *Afzeliaxylocarpa* from Thailand.

###### ﻿Phylogenetic analysis of Hypoxylaceae

**Figure 3.**
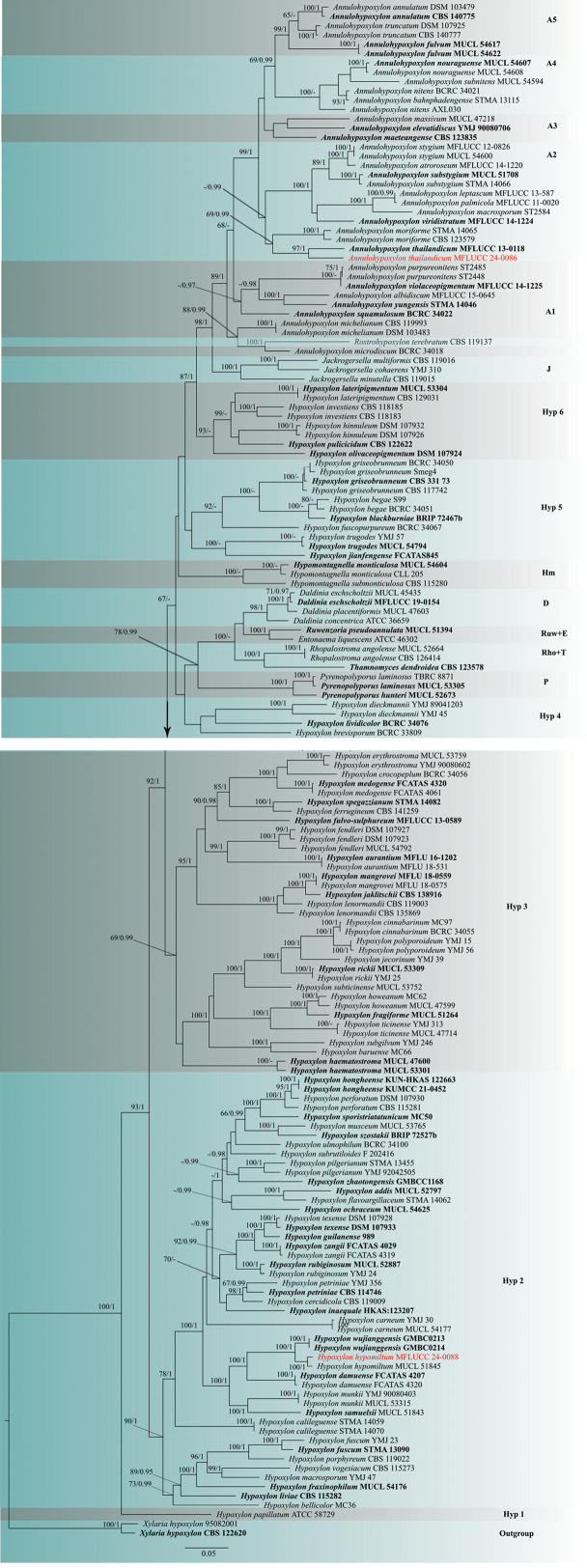
RAxML tree based on the analysis of the combined ITS, LSU, *rpb2*, and *tub2* dataset. ML bootstrap supports (MLBS) equal to or higher than 65%, and the Bayesian posterior probabilities (BYPP) equal to or greater than 0.90 are given near the nodes. The ex-types are in bold. The two new sequences are shown in red font. The phylogenetic analyses constituted *Xylariahypoxylon* (Outgroup) and 11 genera of Hypoxylaceae: *Annulohypoxylon* (A), *Daldinia* (D), *Entonaema* (E), *Hypomontagnella* (Hm), *Hypoxylon* (Hyp), *Jackrogersella* (J), *Pyrenopolyporus* (P), *Rhopalostroma* (Rho), *Rostrohypoxylon* (Ros), *Ruwenzoria* (Ruw) and *Thamnomyces* (T).

The combined phylogenetic dataset of ITS, LSU, *rpb*2, and *tub2* contains 164 isolates belonging to 116 Hypoxylaceae taxa (Suppl. material 1: table S2). After trimming, the analyzed dataset comprised 3,499 characters, including gaps (ITS = 605 bp, LSU = 740 bp, *rpb*2= 1,082 bp, *tub2* = 1,072 bp). The final ML optimization likelihood value of the best RAxML tree was -76207.411440 (Fig. 3), and the matrix had 1933 distinct alignment patterns, with 33.77% undetermined characters or gaps. In our phylogenetic tree, we have labeled the clades as follows: *Annulohypoxylon*: A, *Daldinia*: D, *Entonaema*: E, *Hypomontagnella*: Hm, *Hypoxylon*: Hyp, *Jackrogersella*: J, *Pyrenopolyporus*: P, *Rhopalostroma*: Rho, *Rostrohypoxylon*: Ros, *Ruwenzoria*: Ruw and *Thamnomyces*: T. The topologies of phylogenetic trees based on the combined dataset generated from ML and BI analyses were almost identical, while the statistical supports showed slight differences. Our phylogenetic tree is nearly identical to previously published studies (Wendt et al. 2018; Becker et al. 2020; Lambert et al. 2021; Ma et al. 2022; Cedeño-Sanchez et al. 2023). *Hypoxylonpapillatum* clustered at a basal position (Hyp1) with 100% ML bootstrap support and 1.00 posterior probability support. The clade Hyp2 was segregated from the backbone of the tree with 90% ML bootstrap support and 1.00 posterior probability support. Members of Hyp3 were clustered with 69% ML bootstrap support and 0.99 posterior probability support, while the sister clade Hyp4 clustered with 67% ML bootstrap support. All the clades of *Annulohypoxylon* (A1-A5), *Hypomontagnella*, *Jackrogersella*, *Rostrohypoxylon*, *Pyrenopolyporus*, *Entonaema*, *Ruwenzoria*, *Thamnomyces*, *Rhopalostroma*, and *Daldinia* displayed well-supported segregation in the phylogeny (Fig. 3). Our strain of *A.thailandicum* (MFLUCC 24-0086) clustered closer to *A.thailandicum* (MFLUCC 13-0118) with 97% ML bootstrap support and 1.00 posterior probability support. Our collection (MFLUCC 24-0088) of *H.hypomiltum* clustered with *H.hypomiltum* (MUCL 51845) with 100% ML bootstrap support and 1.00 posterior probability support.

#### ﻿ Annulohypoxylon thailandicum

Taxon classificationFungiXylarialesHypoxylaceae

Daranag. & K.D. Hyde, Fungal Diversity 72: 53 (2015)

27A42BBF-2BC8-5D06-9AA1-BA34FD3217F1

Index Fungorum: IF550799

Facesoffungi Number: FoF00373

Figs 3 , 4

##### Description.

***Saprobic*** on a dead branch of *Swieteniamacrophylla* (Meliaceae). ***Sexual morph***: ***Ascostromata*** 0.8–2 × 0.5–1.5 mm (x–= 1.5 ×1 mm, n = 15), spherical to hemispherical, superficial, effused-pulvinate, conglomerate, solitary or catenated, with conspicuous perithecial mounds, carbonaceous, black surface, blackish granules beneath surface and between perithecia, releasing greenish-olivaceous pigments in 10% KOH. ***Perithecia*** 0.35–0.8 × 0.4–1.0 mm (x– = 0.5 × 0.6 mm, n =15), immersed, spherical to pyriform, encased in carbonaceous tissue. ***Ostioles*** coarsely papillate, truncatum-type disc, 0.25–0.4 mm diam. ***Paraphyses*** not observed. ***Asci*** 79–100 × 3.5–4.9 μm (x– = 91 × 4.9 μm, n = 20), unitunicate, cylindrical, uniseriate, rarely overlapping uniseriate, 8-spored, apical apparatus faintly bluing in Melzer’s reagent. ***Ascospores*** 7–10 × 3.5–5.0 μm (x– = 8.5 × 4.5 μm, n =30), unicellular, ellipsoid-inequilateral, narrowly to broadly rounded ends, hyaline at immaturity, olivaceous brown to brown at maturity, straight spore-length germ slit, epispore smooth, perispore dehiscent in 10% KOH. ***Asexual morph***: Undetermined.

**Figure 4.**
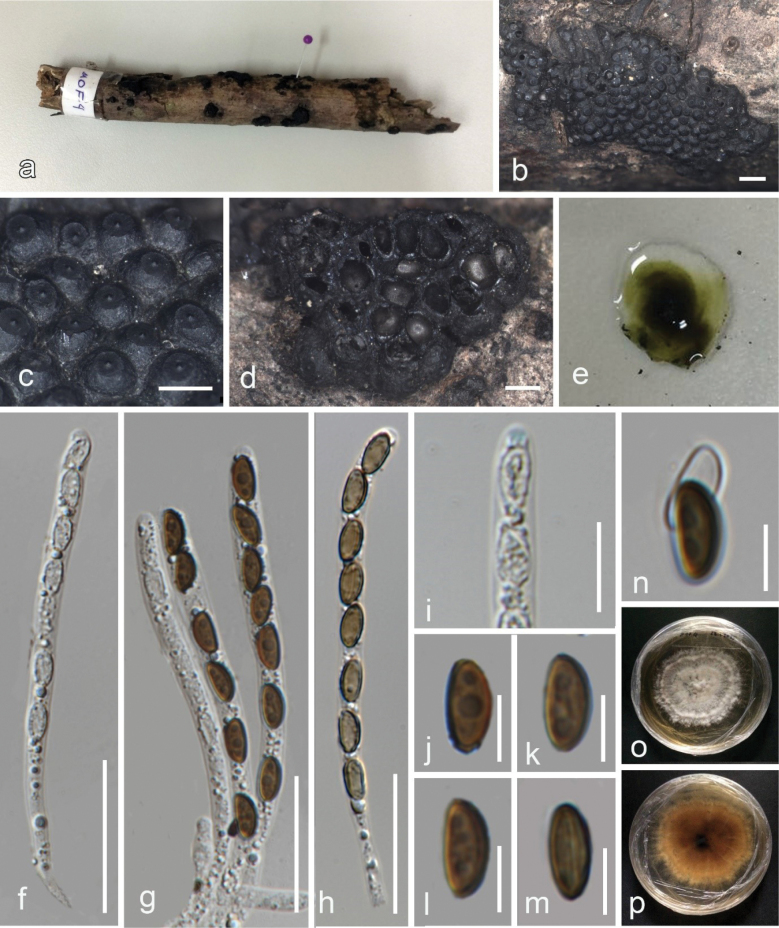
*Annulohypoxylonthailandicum* (MFLU 24-0019, new host record) **a** host **b** stromatal habit on host **c** ostioles with ostiolar discs **d** stromata in horizontal section showing perithecia **e** pigments in KOH **f–h** asci **i** ascal apical apparatus in Melzer’s reagent **j–m** ascospores **o, p** colonies on PDA after two weeks. Scale bars: 1 mm (**b**); 500 µm (**c, d**); 30 µm (**f–h**); 10 µm (**i**); 5 µm (**j–m**).

##### Culture characters.

Colonies on PDA reaching 4 cm after one week, white, effuse, erose, medium, dull, rhizoid edge, no sporulation, top white, reverse white at first, turning light brown after few days (4–5 days).

##### Material examined.

Thailand, Mae Fah Luang University, Chiang Rai, on a dead branch of *Swieteniamacrophylla* (Meliaceae), 20 November 2022, Raheleh Asghari MOF4 (MFLU 24-0019), living culture MFLUCC 24-0086.

##### Known distribution.

China (Zhang et al. 2023), Indonesia (Yurnaliza et al. 2021), and Thailand (Liu et al. 2015, this study).

##### Known hosts.

*Elaeisguineensis* (Yurnaliza et al. 2021) and *Swieteniamacrophylla* (This study).

##### Notes.

Our collection (MFLU 24-0019) resembles *A.thailandicum* in having effused-pulvinate black ascostromata, releasing pigment in KOH, papillate perithecia, with truncatum-type disc, cylindrical asci with J+ apical ring, ellipsoid-inequilateral brown ascospores with germ slit and dehiscent perispore in 10% KOH as detailed in Liu et al. (2015). Our specimen differs slightly in having larger perithecia and smaller ascospores (Table 2). Sharing similar morphological characteristics, such as ascospores, ostiolar disc, and the release of pigments in KOH, *A.thailandicum* (MFLU 24-0019) can be compared to *A.archeri* and *A.microcarpum* (Liu et al. 2015). However, it is crucial to note that the latter two species exhibit reddish ascostromata with a smaller truncatum-type disc, distinguishing them from *A.thailandicum*. In the multi-loci phylogenetic tree, *A.thailandicum* isolate MFLUCC 24-0086 clustered with *A.thailandicum* (MFLUCC 13-0118) with 100% ML bootstrap support and 1.00 posterior probability support (Fig. 3). In this study, we report our collection (MFLU 24-0019) as a new host record on *Swieteniamacrophylla* from Thailand.

**Table 2.** Synopsis of morphological characteristics of *Annulohypoxylonthailandicum*, *A.archeri*, and *A.microcarpum*.

Name	stromata size (mm)	Perithecia (mm)	Asci (μm)	Apical ring (μm)	Ascospore size (μm)	Germ slit
*A.thailandicum*MFLU 13-0441 (Liu et al. 2015)	0.5–0.7 × 0.8–1.7 × 0.8 (x–=0.7 × 1.5 × 0.8)	0.35–0.5 × 0.3–0.7 (x–=0.4 × 0.5)	89–100.8 × 4.5–6.5 (x–=97 × 5.6)	globose, 1.5 × 2	6–11.5 × 4–6.5 (x–=12.5 × 5.6)	Straight
*A.thailandicum*MFLU 24-0019 (This study)	0.8–2 × 0.5–1.5 (x–= 1.5 × 1)	0.35–0.8 × 0.4–1.0 mm (x– = 0.5 × 0.6 mm)	79–100 × 3.5–4.9 μm (x– = 91 × 4.9 μm)	discoid, wedge, 1.4–1.8 × 0.7–1.1	7–10 × 3.5–5.0 μm (x– = 8.5 × 4.5 μm)	Straight
*A.archeri* (Ju and Rogers 1996)	–	0.1 mm diam	–	–	9–10.5 μm × 4–5 μm	–
*A.microcarpum* (Ju and Rogers 1996)	–	0.15–0.2 mm diam	–	–	7–8 × 3–4μm	–

#### ﻿ Hypoxylon hypomiltum

Taxon classificationFungiXylarialesHypoxylaceae

Mont., Annls Sci. Nat., Bot., sér. 2 13: 356 (1840)

66701ACC-1CCA-5BC4-9A54-0C43E296F7EF

Index Fungorum: IF158066

Facesoffungi Number: FoF06137

Figs 3 , 5

##### Description.

***Saprobic*** on dead wood of *Dalbergiacana* (Fabaceae). ***Sexual morph***: ***Stromata*** superficial, sessile, effused, pulvinate, conspicuous, hemispherical, dense, forming compact mass, discoid, black, KOH extractable pigments yellow. ***Perithecia*** 360–400 × 300–350 μm (x– = 350 × 300 µm, n =15), hemispherical to spherical. ***Asci*** cylindrical, 8-spored, uniseriate, spore-bearing part 43–55 × 4–6 μm (x– = 48 × 5 μm, n = 20), and stipes 37–50 (x– = 45, n = 20), with amyloid apical apparatus bluing in Melzer’s reagent, discoid, 0.4–0.6 × 1.3–1.5 µm (x– = 5 × 1.4 μm, n = 10). ***Ascospores*** 6–8 × 3–4 µm (x– = 7 × 3.5 µm n = 20), brown, ellipsoidal with rounded ends, uniseriate, aseptate, guttulate, straight germ slit. ***Asexual morph***: Undetermined.

##### Culture characters.

Colonies grown on PDA, reaching 55 mm in diameter after 20 days at 25 °C, under dark conditions, circular, flat, smooth, entire edge, medium dense, mycelium superficial to immersed, no sporulation, yellow pigment diffusion, light brown on the top and reverse pale yellowish-brown.

##### Material examined.

Thailand, Doi Tung National Park, Chiang Rai, on dead wood of *Dalbergiacana* (Fabaceae), 27 September 2022, N. Afshari 4C3T2R3a (MFLU 24-0043), living culture MFLUCC 24-0088.

**Figure 5.**
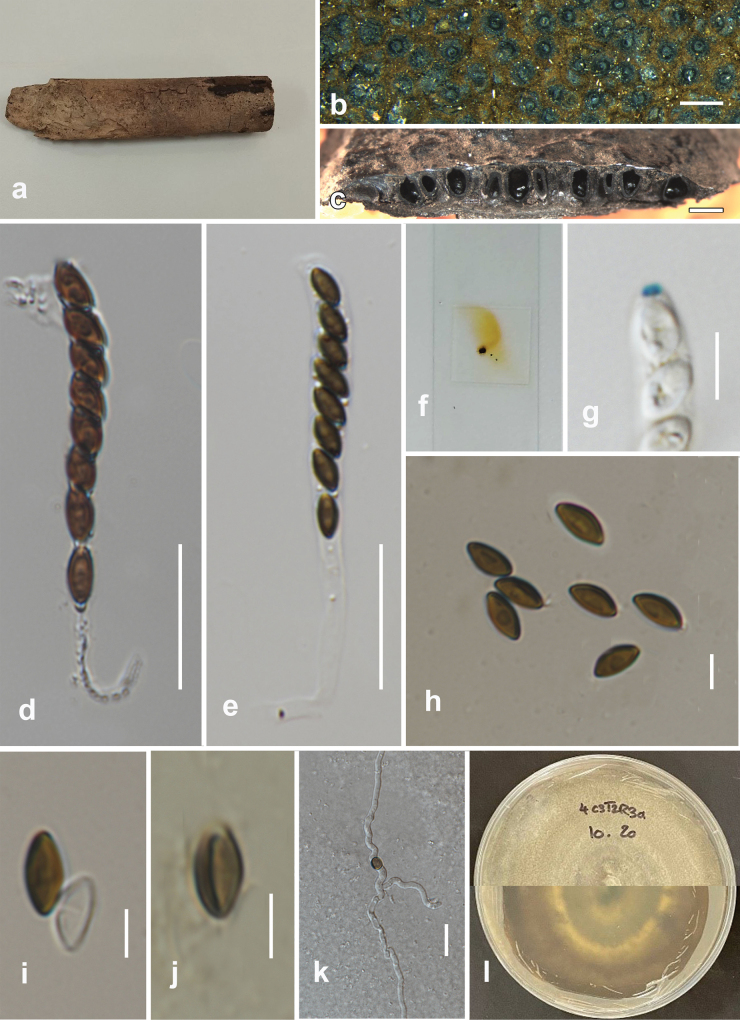
*Hypoxylonhypomiltum* (MFLU 24-0043, new host and geographical record) **a** host **b** stromatal habit on host **c** stromata in vertical section showing perithecia **d, e** asci **f** pigments in KOH **g** ascal apical apparatus in Melzer’s reagent **h-i** ascospore (**i** ascospore with perispore **j** ascospore with germ slit) **k** germinated ascospore **l** colony on PDA after three weeks. Scale bars: 500 µm (**b, c**); 20 µm (**d, e**); 5 µm (**g-j**); 10 µm (**k**).

##### Known distribution.

French Guiana (Montagne 1840), Japan (Abe 1986), Panama (Standley 1933), Pakistan (Ahmad 1978), Sri Lanka (Palapathwala et al. 2019), Thailand (This study), USA (Ju and Rogers 1996), and India (Sarbhoy et al. 1971).

##### Known hosts.

*Faguscrenata* (Abe 1986), *Morusalba* (Ahmad 1978), *Mangiferaindica* (Sarbhoy et al. 1971), and *Dalbergiacana* (This study).

##### Notes.

Our collection (MFLU 24-0043) shares similar characteristics with *Hypoxylonhypomiltum* (Montagne 1840), including effuse, pulvinate, conspicuous, hemispherical stromata, KOH extractable pigments, hemispherical to spherical perithecia, cylindrical, uniseriate asci, brown, ellipsoidal, unicellular ascospores with a straight germ slit, and mostly similar sized stromata, perithecia, asci and ascospores. In the phylogenic analyses (Fig. 3), our collection (MFLUCC 24-0088) clustered with *Hypoxylonhypomiltum* (MUCL52887) with 100% ML bootstrap support and 1.00 posterior probability support. In this study, we report our isolate (MFLU 24-0043) as a new host and geographical record of *Hypoxylonhypomiltum* on *Dalbergiacana* from Thailand.

###### ﻿Phylogenetic analysis of Oxydothidaceae

**Figure 6.**
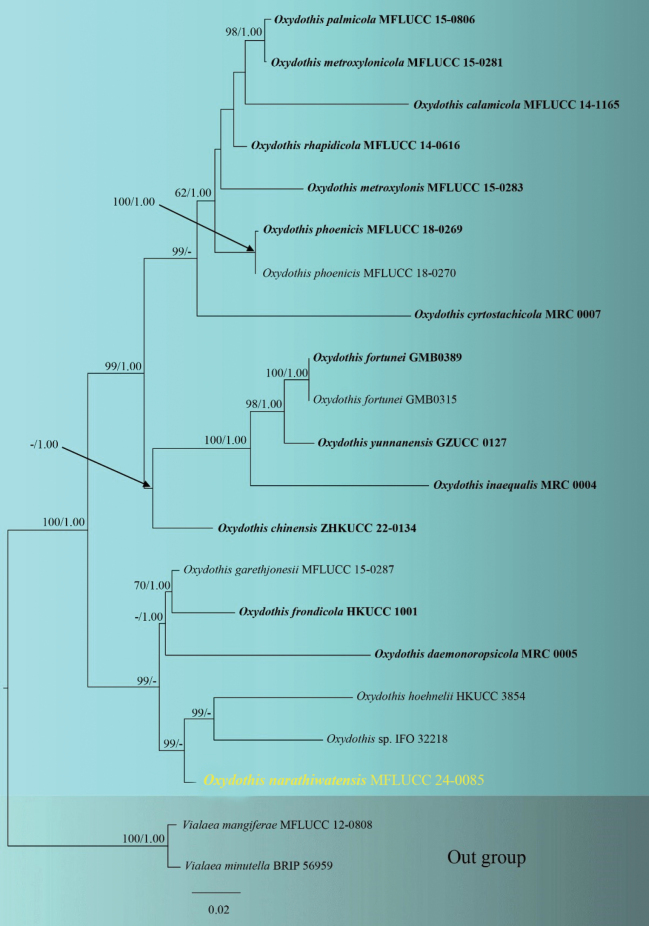
RAxML tree is based on the analysis of a combined dataset of ITS, LSU, and SSU sequence data. Maximum likelihood bootstrap support (MLBS) values equal to or higher than 60%, and the Bayesian posterior probability (BYPP) equal to or greater than 0.95 are given near the nodes. The ex-types are in bold. The new sequence is shown in yellow font. The tree is rooted with *Vialaeamangiferae* and *Vialaeaminutella*.

The combined ITS, LSU, and SSU dataset consisted of 20 isolates belonging to Oxydothidaceae taxa, with *Vialaeamangiferae* (MFLUCC 12-0808) and *Vialaeaminutella* (BRIP 56959) as outgroup taxa (Suppl. material 1: table S3). The final alignment comprised 2,657 characters (ITS: 452 bp, LSU: 1,200 bp, SSU: 1,021 bp), including gaps. The final ML optimization likelihood value of the best RaxML tree was -9733.301849 (Fig. 6), and the matrix had 1,637 distinct alignment patterns, with 30% undetermined characters or gaps. Estimated base frequencies were as follows: A = 0.256773, C = 0.222793, G = 0.273403, T = 0.247031; substitution rates AC = 1.198924, AG = 2.053309, AT = 1.196302, CG = 1.103201, CT = 3.773909, GT = 1.000000; gamma distribution shape parameter α = 0.253842. The topology of our phylogenetic tree is identical to previous publications (Konta et al. 2016; Hu et al. 2022; Senanayake et al. 2023). Our collection of the new species *Oxydothisnarathiwatensis* (MFLUCC 24-0085) is from a distinct clade with *Oxydothishoehnelii* (HKUCC 3854) and *Oxydothis* sp. (IFO 32218) with 99% ML bootstrap support (Fig. 6).

#### ﻿ Oxydothis narathiwatensis

Taxon classificationFungiXylarialesOxydothidaceae

O. Karimi & K.D. Hyde sp. nov.

FA52FE61-398A-5E60-BC7C-4A08B43D4F6A

Index Fungorum: IF902133

Facesoffungi Number: FoF16036

Figs 6 , 7 , 8

##### Etymology.

The epithet “*narathiwatensis*” refers to Narathiwat Province, where the holotype was collected.

##### Holotype.

MFLU 24-0044.

##### Description.

***Saprobic*** on the submerged rachis of *Eleiodoxaconferta* (Arecaceae). ***Sexual morph***: ***Ascomata*** 170–320 μm diam., (x– = 250 μm diam., n = 15), mostly in small groups, immersed, erumpent, with the non-blistering area on the host, subglobose or pyriform. ***Peridium*** 17–30 μm (x– = 22 μm, n = 10), thick, dark brown to black, textura angularis. ***Paraphyses*** 40–80 × 3–6 μm (x– = 62 × 4 μm, n = 20), cylindrical, fragmented, hyaline, branched or non-branched. ***Asci*** 171–257 × 7–11 μm (x– = 225 × 9 μm, n = 20), 8-spored, cylindrical, unitunicate, short pedicellate, smooth-walled, with a J+, wedge-shaped, subapical ring. ***Ascospores*** 95–121 × 3–5 μm (x– = 110 × 4 μm, n = 20), 2–3-seriate, hyaline, filiform, straight, curved or flexuous, rounded ends, centrally uniseptate, guttulate with smooth walls. ***Appressoria*** 10–20 × 9–10 μm (x– = 13 × 9.5 μm, n = 10), irregular, hyaline to green, thick-walled, verrucose. ***Asexual morph***: Undetermined.

##### Culture characters.

Colonies on PDA, reaching 55 mm in diameter after 30 days at 25–27 °C, under dark conditions, medium dense, mycelium superficial to immersed, circular, flat, raised in the center with aerial mycelium, dull surface, entire edge, velvety, without pigment diffusion and sporulation, dark brown on the top and reverse-side black.

##### Material examined.

Thailand, Narathiwat, peat swamp forest, on the submerged rachis of *Eleiodoxaconferta* (Arecaceae), 3 August 2023, O. Karimi, 19-W (MFLU 24-0044, holotype); Ex-type living culture MFLUCC 24-0085.

##### Notes.

**Figure 7.**
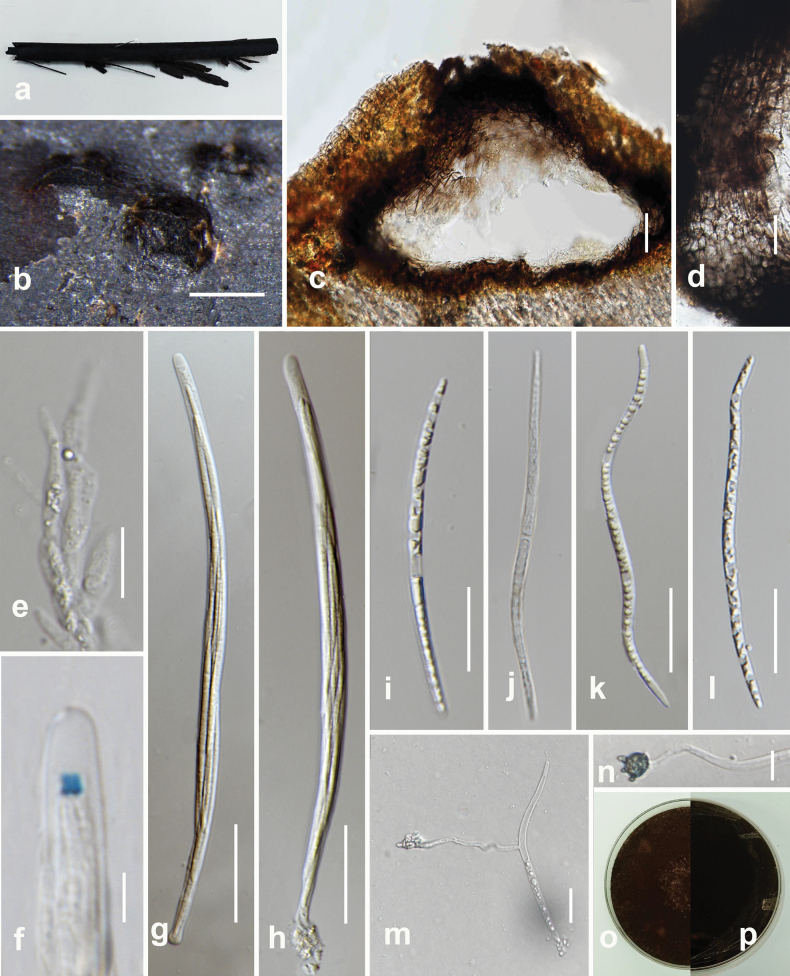
*Oxydothisnarathiwatensis* (MFLU 24-0044, holotype) **a** host substrate **b** close up of ascomata **c** section of ascoma **d** peridium **e** paraphyses **f** j+ reaction of apical ring in Melzer’s reagent **g, h** asci **i–l** ascospores **m** germinating ascospore **n** appressoria **o, p** colony on PDA after two weeks. Scale bars: 500 μm (**b**); 50 μm (**c, d, g, h**); 20 μm (**e, m**); 5 μm (f); 25 μm (**i–l**); 10 μm (**n**).

**Figure 8.**
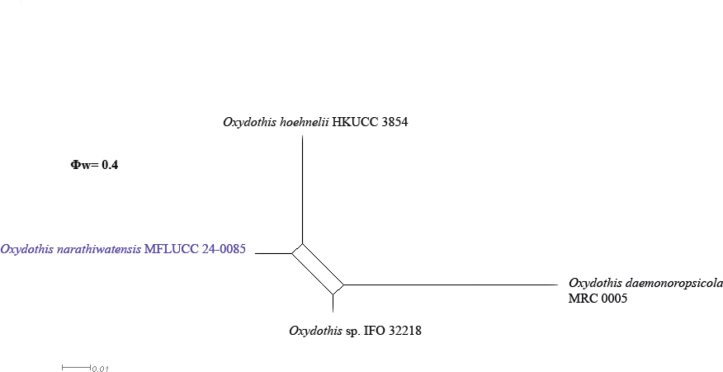
The split diagram resulting from the pairwise homoplasy index (PHI) test was constructed using the combined ITS, LSU, and SSU sequence data of closely related taxa. The PHI test (Φw) < 0.05 indicates significant recombination within the dataset. The newly identified taxon is represented in blue.

Morphologically, *Oxydothisnarathiwatensis* (MFLU 24-0044) shares similar characteristics with *O.gigantea* (BRIP 21921) and *O.maquilingiana* (3975) in having cylindrical asci with J+, wedge-shaped, subapical ring and filiform ascospores (Hyde 1994b). However, *O.narathiwatensis* (MFLU 24-0044) differs from *O.gigantea* (BRIP 21921) in having longer and narrower asci (171–257 × 7–11 μm vs. 240 × 20 µm), and shorter and narrower ascospore (95–121 × 3–5 μm vs. 100–150 × 6.5–7.5 µm). *Oxydothisnarathiwatensis* (MFLU 24-0044) differs from *O.maquilingiana* (3975) in having longer and narrower asci (171–257 × 7–11 μm vs. 140–150 × 12–14 µm), longer and narrower ascospore (95–121 × 3–5 μm vs. 85–95 × 5–6 µm) and longer ascal ring (1.5–5 × 1–3 µm vs. 2.6–3.5 × 1.6–2.4 µm). However, due to the lack of sequence data for *O.gigantea* and *O.maquilingiana*, a phylogenetic comparison with *O.narathiwatensis* was not possible. Phylogenetically, *O.narathiwatensis* (MFLUCC 24-0085) formed a robust subclade (100% ML) basal to *O.hoehnelii* (KDH 1837). Morphologically, *O.narathiwatensis* differs from *O.hoehnelii* in having shorter and narrower asci (171–257 × 7–11 μm vs. 250–290 × 12–14 µm), fusiform ascospores against filiform ascospores in *O.narathiwatensis* (MFLU 24-0044) and longer and narrower ascospores (95–121 × 3–5 μm vs. 72–86 × 7–10 μm). The result of the pairwise homoplasy index (PHI) test revealed no significant recombination (Φw = 0.4) between *O.narathiwatensis* (MFLUCC 24-0085) and its closely related species (Fig. 8). Therefore, we introduced *Oxydothisnarathiwatensis* (MFLU 24-0044) as a novel species based on morphological evidence and phylogenetic analyses (Figs 6–8).

## ﻿Discussion

In this study, xylariales-like specimens were collected from Doi Tung National Park and Mae Fah Luang University in Chiang Rai Province, located in northern Thailand, and from Narathiwat Province in southern Thailand. Our investigation, employing a polyphasic approach that combines both morphological and molecular analyses (Figs 1–8), resulted in the identification of one novel species, *Oxydothisnarathiwatensis*, and documented a new host record for *Annulohypoxylonthailandicum* from *Swieteniamacrophylla* and two new host and geographical records of *Xylariabawanglingensis* and *Hypoxylonhypomiltum* from *Afzeliaxylocarpa* and *Dalbergiacana*, respectively, in Thailand.

Our *Xylaria* species were collected from the dead wood of *Afzeliaxylocarpa*, and phylogenetic analysis reveals that it forms a sister clade with the wood-inhabiting species, *Xylariabawanglingensis* (GMB1023 and GMB1162) and *Xylariafeejeensis* (HAST 92092013 and JRD 180). However, *Xylariaphyllocharis* (HAST 528), collected on fallen leaves (Dennis 1956) is separated from these two wood-inhabiting species in our phylogenetic tree; this finding agrees and is comparable with previous studies (Hsieh et al. 2010; Pan et al. 2022).

*Annulohypoxylonthailandicum* (MFLU 13-0441) was described by Daranagama & K.D. Hyde in Liu et al. (2015) from Chiang Mai. In the phylogenetic analyses, the closest species to our isolate (MFLUCC 24-0086) was found to be *A.thailandicum* (MFLUCC 13-0118), with slight differences in the ascostromata size. The relatedness is also statistically well-supported in our phylogenic analyses (Fig. 3). *Annulohypoxylonthailandicum* (MFLU 13-0441) has also been reported from Guizhou, China (Zhang et al. 2023), and North Sumatra, Medan City, Indonesia (Yurnaliza et al. 2021). The climate of Doi Suthep, Chiang Mai, Chiang Rai (north of Thailand), Guizhou (China), and Medan City, North Sumatra (Indonesia) are mostly similar, showing a preference for Hypoxylaceae for tropical to subtropical climates. Hence, we believe that conducting more studies in tropical to subtropical climatic areas will enhance our understanding and lead to the discovery of more undescribed species of *Annulohypoxylon*.

The delimiting characteristics of the sexual morph of *Hypoxylon* include never erecting unipartite stromata, with solid and homogenous basal tissue underneath the perithecial layer (Ju and Rogers 1996). Based on the morphological assessment, our collection fits well with *Hypoxylonhypomiltum* (Montagne 1840). In our phylogenetic analysis (Fig. 3), there is a notable clustering of all four isolates of *H.griseobrunneum*, which diverges from the findings in recent publications (Ma et al. 2022; Song et al. 2022; Yang et al. 2022). This discrepancy may stem from the utilization of sequences with incorrect names. For instance, Hsieh et al. (2005) provided *β-tubulin* and *actin* sequences for the *Hypoxylonanthochroum* isolate BCRC 34050 and incorporated them into their phylogenetic tree. However, Kuhnert et al. (2015) later updated the status of the BCRC 34050 isolate, assigning it to *H.griseobrunneum* after conducting morphological and phylogenetic analyses that revealed a close relationship with *H.griseobrunneum* isolates. Subsequently, this sequence was employed for the latter species. In contrast, recent publications (Ma et al. 2022; Song et al. 2022; Yang et al. 2022) have utilized the sequence of the BCRC 34050 isolate as *H.anthochroum*. Therefore, due to frequent changes in taxonomy, it is crucial to carefully choose accurate sequences and species names.

*Oxydothis* species belong to the family Oxydothidaceae and comprise approximately 80 species, predominantly associated with palms (Arecaceae) in various habitats, including peat swamps, marine, and terrestrial environments, where they are primarily found as saprobes (Hyde 1993a, 1993b, 1994b; Wang and Hyde 1999, 2001; Fröhlich and Hyde 2000; Taylor and Hyde 2003; Shenoy et al. 2005; Hidayat et al. 2006). Despite the substantial diversity within this genus, only 15 species have sequence data. Among these, some species lack crucial genetic markers, such as ITS sequences (e.g., *O.calamicola*, and *O.rhapidicola*) or SSU or LSU sequences (e.g., *O.chinensis*, *O.cyrtostachicola*, and *O.fortunei*), which impacts the accuracy of phylogenetic reconstructions (Hidayat et al. 2006; Konta et al. 2016; Hu et al. 2022; Senanayake et al. 2023). Therefore, obtaining sequence data for previously described species is imperative to enhance our understanding of the phylogenetic relationships among *Oxydothis* species. In our study, we proposed a new species collected from submerged rachides of *Eleiodoxaconferta* in Narathiwat Province. We provided sequence data (ITS, LSU, and SSU) with detailed morphological descriptions. Notably, our investigation revealed the production of appressoria by germinating ascospores in our saprobic species, *O.narathiwatensis*, is similar to *O.garethjonesii*, *O.metroxylonicola*, *O.metroxylonis*, and *O.palmicola* (Konta et al. 2016). Appressoria in saprobic fungi raises intriguing questions about their ecological role and evolutionary adaptation. Some *Oxydothis* species are endophytes, and several produce appressoria, indicating an endophytic lifestyle (Taylor et al. 1999; Konta et al. 2016). This may suggest that these species are likely host-specific endophytes that change their lifestyle and become early saprobes when palm fronds die (Chethana et al. 2021). This supports the hypothesis that many saprobes initially start as endophytes (Chethana et al. 2021; Bhunjun et al. 2024). Additionally, the production of appressoria can help the fungi attach to their substrate in aquatic habitats (Au et al. 1996). Further exploration into the functional role of appressoria in *Oxydothis* species is warranted and could yield valuable insights into their ecology, interactions with host plants, and broader evolutionary strategies. This research has the potential to significantly enhance our understanding of fungal ecology and evolution in diverse ecosystems, shedding light on the intricate relationships between their host plants.

## Supplementary Material

XML Treatment for Xylaria bawanglingensis

XML Treatment for Annulohypoxylon thailandicum

XML Treatment for Hypoxylon hypomiltum

XML Treatment for Oxydothis narathiwatensis

## ﻿Additional information

### Author ORCIDs

Omid Karimi https://orcid.org/0000-0001-9652-2222

Naghmeh Afshari https://orcid.org/0009-0000-0740-6327

Raheleh Asghari https://orcid.org/0009-0006-4897-5327

Qirui Li https://orcid.org/0000-0001-8735-2890

K. W. Thilini Chethana https://orcid.org/0000-0002-5816-9269

Kevin D. Hyde https://orcid.org/0000-0002-2191-0762

Fatimah O. Alotibi https://orcid.org/0000-0003-3629-5755

### Data availability

All of the data that support the findings of this study are available in the main text or Supplementary Information.

## Supplementary materials

Supplementary material 1 Supplementary information Data type docx Explanation note **table S1.** List of taxa used for the phylogenetic reconstruction (Xylariaceae). GenBank accession numbers, specimen number, origin, status and reference. **table S2.** List of taxa used for the phylogenetic reconstruction (Hypoxylaceae). **table S3.** List of taxa used for the phylogenetic reconstruction (Oxydothidaceae). https://binary.pensoft.net/file/1110125This dataset is made available under the Open Database License (http://opendatacommons.org/licenses/odbl/1.0/). The Open Database License (ODbL) is a license agreement intended to allow users to freely share, modify, and use this Dataset while maintaining this same freedom for others, provided that the original source and author(s) are credited.Omid Karimi, Naghmeh Afshari, Raheleh Asghari, Qirui Li, K. W. Thilini Chethana, Kevin D. Hyde, Fatimah O. Alotibi
