# Supplementary material for: ﻿Novel discoveries of Xylariomycetidae (Ascomycota) taxa from peat swamp forests and other terrestrial habitats in Thailand
Source: MycoKeys. 2024 Aug 7;107:219–47. doi: 10.3897/mycokeys.107.127749 (PMC11336385; doi:10.3897/mycokeys.107.127749)
Supplement: Supplementary material 1 — Supplementary information [file mycokeys-107-219-s001.docx]

**Supplementary Material 1**

**Table S1.** List of taxa used for the phylogenetic reconstruction (Xylariaceae). GenBank accession numbers, specimen number, origin, status and reference. Newly generated sequences are in **bold,** and the type strains are marked with *. “_” Sequences were not available.

| Taxon | **Culture collection/ Voucher no**. | **ITS** | ***rpb*2** | ***tub*** | ***act*** |
| --- | --- | --- | --- | --- | --- |
| *Amphirosellinia fushanensis** | HAST 91111209 | GU339496 | GQ848339 | GQ495950 | GQ452360 |
| *Astrocystis bambusae* | HAST 89021904 | GU322449 | GQ844836 | GQ495942 | GQ449239 |
| *Astrocystis concavispora** | MFLUCC 14-0174 | KP297404 | KP340532 | KP406615 | _ |
| *Astrocystis mirabilis** | HAST 94070803 | GU322448 | GQ844835 | GQ495941 | GQ449238 |
| *Astrocystis sublimbata* | HAST 89032207 | GU322447 | GQ844834 | GQ495940 | GQ449236 |
| *Brunneiperidium gracilentum** | MFLUCC 14-0011 | KP297400 | KP340528 | KP406611 | _ |
| *Collodiscula bambusae** | GZUH0102 | KP054279 | KP276675 | KP276674 | _ |
| *Collodiscula fangjingshanensis** | GZUH0109 | KR002590 | KR002592 | KR002589 | _ |
| *Collodiscula leigongshanensis** | GZUH0107 | _ | KR002588 | KR002587 | _ |
| *Daldinia loculatoides** | CBS:113279 | MH862918 | KY624247 | KX271246 | _ |
| *Entoleuca mammata** | JDR 100 | GU300072 | GQ844782 | GQ470230 | _ |
| *Hypocreodendron sanguineum** | JDR 169 | GU322433 | GQ844819 | GQ487710 | _ |
| *Hypoxylon fragiforme** | MUCL 51264 | KC477229 | KM186296 | KX271282 | _ |
| *Hypoxylon monticulosum** | MUCL 54604 | KY610404 | KY624305 | KX271273 | _ |
| *Kretzschmaria deusta* | CBS 163.93 | KC477237 | KY624227 | KX271251 | _ |
| *Kretzschmaria guyanensis** | HAST 89062903 | GU300079 | GQ844792 | GQ478214 | GQ408901 |
| *Kretzschmariella culmorum* | JDR 88 | KX430043 | KX430045 | KX430046 | _ |
| *Nemania abortiva** | 467 BISH | GU292816 | GQ844768 | GQ470219 | _ |
| *Nemania beaumontii* | 405 (HAST, JF) | GU292819 | GQ844772 | GQ470222 | _ |
| *Nemania bipapillata* | HAST 90080610 | GU292818 | GQ844771 | GQ470221 | GQ389693 |
| *Nemania primolutea** | HAST 91102001 | EF026121 | GQ844767 | EF025607 | EF025592 |
| *Neoxylaria arengae** | MFLUCC 15-0292 | MT496747 | MT502418 | _ | _ |
| *Podosordaria mexicana** | 176 WSP | GU324762 | GQ853039 | GQ844840 | _ |
| *Podosordaria muli** | 167 WSP | GU324761 | GQ853038 | GQ844839 | _ |
| *Poronia pileiformis** | 88113001 WSP | GU324760 | GQ853037 | GQ502720 | GQ455449 |
| *Poronia punctata* | CBS 656.78 | KT281904 | _ | KX271281 | _ |
| *Rosellinia aquila* | MUCL 51703 | KY610392 | KY624285 | KX271253 | _ |
| *Rosellinia buxi** | JDR 99 | GU300070 | GQ844780 | GQ470228 | GQ398228 |
| *Rosellinia corticium* | MUCL 51693 | KY610393 | KY624229 | KX271254 | _ |
| *Rosellinia merrillii** | HAST 89112601 | GU300071 | GQ844781 | GQ470229 | GQ398229 |
| *Stilbohypoxylon elaeidicola* | Y.M.J. 173 | EF026148 | GQ844826 | EF025616 | _ |
| *Stilbohypoxylon elaeidis** | MFLUCC 15-0295a | MT496745 | MT502416 | MT502420 | _ |
| *Stilbohypoxylon elaeidis* | MFLUCC 15-0295b | MT496746 | MT502417 | MT502421 | _ |
| *Xylaria acuminatilongissima** | HAST 623 | EU178738 | GQ853028 | GQ502711 | GQ853046 |
| *Xylaria adscendens* | HAST 570 | GU300101 | GQ844817 | GQ487708 | GQ438745 |
| *Xylaria allantoidea* | HAST 94042903 | GU324743 | GQ848356 | GQ502692 | GQ452377 |
| *Xylaria amphithele* | HAST 529 | GU300083 | GQ844796 | GQ478218 | GQ408905 |
| *Xylaria apoda* | HAST 90080804 | GU322437 | GQ844823 | GQ495930 | GQ438751 |
| *Xylaria arbuscula* | HAST 89041211 | GU300090 | GQ844805 | GQ478226 | GQ421286 |
| *Xylaria arbuscula var plenofissura* | HAST 93082814 | GU339495 | GQ844804 | GQ478225 | GQ421285 |
| *Xylaria atrodivaricata******** | HAST 95052001 | EU178739 | GQ853030 | GQ502713 | GQ853048 |
| *Xylaria atrosphaerica* | HAST 91111214 | GU322459 | GQ848342 | GQ495953 | GQ452363 |
| *Xylaria badia* | HAST 95070101 | GU322446 | GQ844833 | GQ495939 | GQ449235 |
| *Xylaria bambusicola* | JDR 162 | GU300088 | GQ844801 | GQ478223 | GQ408910 |
| *Xylaria baoshanensis** | GMB1022 | OR468973 | _ | _ | _ |
| *Xylaria baoshanensis* | GMB1161 | OR468974 | _ | _ | _ |
| *Xylaria bawanglingensis** | GMB1023 | OR468975 | OR753861 | OR477223 | _ |
| *Xylaria bawanglingensis* | GMB1162 | OR468976 | OR753862 | OR477224 | _ |
| ***Xylaria bawanglingensis*** | **MFLUCC 24-0087** | **PP824655** | **PP826252** | **PP826253** | **PP833217** |
| *Xylaria berteri* | JDR 256 | GU324750 | GQ848363 | GQ502698 | GQ455442 |
| *Xylaria berteri* | HAST 90112623 | GU324749 | GQ848362 | AY951763 | AY951874 |
| *Xylaria botryoidalis** | GMB1057 | OR468978 | OR753871 | OR477225 | _ |
| *Xylaria botryoidalis* | GMB1164 | OR468977 | OR753872 | OR477226 | _ |
| *Xylaria brunneovinosa** | HAST 720 | EU179862 | GQ853023 | GQ502706 | GQ853041 |
| *Xylaria castorea* | PDD 600 | GU324751 | GQ853018 | GQ502703 | GQ455447 |
| *Xylaria cf glebulosa* | HAST 431 | GU322462 | GQ848345 | GQ495956 | GQ452366 |
| *Xylaria cf nigripes* | SWUF17-31 2 | MT622790 | _ | MW459229 | MW459202 |
| *Xylaria chaiyaphumensis* | SWUF16-04 1 | MT622777 | _ | _ | _ |
| *Xylaria chaiyaphumensis* | SWUF16-11 4 | MT622776 | _ | MW459236 | MW459213 |
| *Xylaria chaiyaphumensis* | SWUF17-15.1 | MT622774 | _ | _ | MW459214 |
| *Xylaria chaiyaphumensis** | SWUF17-49.2 | MT622775 | _ | _ | MW459215 |
| *Xylaria cirrata* | HAST 664 | EU179863 | GQ853024 | GQ502707 | GQ853042 |
| *Xylaria coccophora* | HAST 786 | GU300093 | GQ844809 | GQ487701 | GQ421289 |
| *Xylaria conica* | SWUF18-4.3 | MT622786 | _ | _ | MW459223 |
| *Xylaria conica** | SWUF18-4.4 | MT622787 | _ | MW459243 | MW459224 |
| *Xylaria coprinicola* | 1145 | HM585020 | HM585019 | HM585018 | HM585017 |
| *Xylaria crinalis* | FCATAS 751 | MF774330 | _ | – | _ |
| *Xylaria crozonensis* | HAST 398 | GU324748 | _ | GQ502697 | GQ455441 |
| *Xylaria cubensis* | HAST 477 | _ | GQ848364 | GQ502699 | GQ455443 |
| *Xylaria culleniae* | JDR 189 | GU322442 | GQ844829 | GQ495935 | GQ438756 |
| *Xylaria dadugangensis** | GMB1036 | OR468979 | OR753863 | OR504178 | _ |
| *Xylaria doupengshanensis** | GMB1037 | OR468980 | OR753864 | OR487773 | _ |
| *Xylaria doupengshanensis* | GMB0773 | OR468981 | OR753865 | OR487774 | _ |
| *Xylaria escharoidea* | HAST 658 | EU179864 | GQ853026 | GQ502709 | GQ853044 |
| *Xylaria fanglanii** | GMB1038 | _ | OR753866 | OR484923 | _ |
| *Xylaria feejeensis* | HAST 92092013 | GU322454 | GQ848336 | GQ495947 | GQ449243 |
| *Xylaria feejeensis* | JRD 180 | GU322453 | GQ848335 | GQ495946 | GQ449242 |
| *Xylaria ficicola* | HMJAU 22818 | MZ351258 | – | – | _ |
| *Xylaria filiformis* | FCATAS 750 | MF774332 | – | – | _ |
| *Xylaria filiformis* | GUM 1052 | KP218907 | – | – | _ |
| *Xylaria fimbriata* | HAST 491 | GU324753 | GQ853022 | GQ502705 | GQ853040 |
| *Xylaria fulvescens** | SWUF17-27 2 | MT622780 | _ | MW459238 | MW459218 |
| *Xylaria glaucae** | GMB1051 | OR468984 | OR753869 | OR484926 | _ |
| *Xylaria glaucae* | GMB1163 | OR468983 | OR753870 | OR484927 | _ |
| *Xylaria grammica* | HAST 479 | GU300097 | GQ844813 | GQ487704 | _ |
| *Xylaria griseosepiacea* | HAST 641 | EU179865 | GQ853031 | GQ502714 | GQ853049 |
| *Xylaria guizhouensis** | GMB1059 | OR468982 | OR753873 | OR484928 | _ |
| *Xylaria guizhouensis* | GMB1058 | OR468986 | OR753874 | OR484929 | _ |
| *Xylaria hedyosmicola** | FCATAS 856 | MZ227121 | MZ683407 | MZ221183 | _ |
| *Xylaria hedyosmicola** | FCATAS 857 | MZ227023 | MZ851780 | MZ221184 | _ |
| *Xylaria hypoxylon* | HAST 152 | GU300096 | GQ844812 | GQ260187 | GQ427196 |
| *Xylaria hypoxylon* | HAST 95082001 | GU300095 | GQ844811 | GQ487703 | GQ427195 |
| *Xylaria hypoxylon* | CBS 122617 | AM993146 | – | – | _ |
| *Xylaria ianthinovelutina* | HAST 553 | GU322441 | GQ844828 | GQ495934 | GQ438755 |
| *Xylaria insolita* | HAST 990903011251 | MN655979 | MN656981 | MN656983 | MN656985 |
| *Xylaria intracolorata* | HAST 90080402 | GU324741 | GQ848354 | GQ502690 | GQ452375 |
| *Xylaria intraflava** | HAST 725 | EU179866 | GQ853035 | GQ502718 | GQ853053 |
| *Xylaria ischnostroma* | SWUF18-22 1 | MT622788 | _ | MW459244 | MW459225 |
| *Xylaria japonica** | GMB1079 | OR468985 | OR887270 | OR485581 | _ |
| *Xylaria japonica* | GMB1080 | OR468987 | _ | OR485582 | _ |
| *Xylaria jinghongensis** | GMB1133 | _ | OR753875 | OR484930 | _ |
| *Xylaria jinshanensis* | GMB1165 | OR468989 | OR753877 | OR484932 | _ |
| *Xylaria jinshanensis** | GMB1067 | OR468988 | OR753876 | OR484931 | _ |
| *Xylaria juruensis* | HAST 92042501 | GU322439 | GQ844825 | GQ495932 | GQ438753 |
| *Xylaria karsticola* | GA1A | MW996752 | _ | _ | _ |
| *Xylaria karsticola** | MUCL 51605 | FN689802 | _ | _ | _ |
| *Xylaria kuankuoshuiensis** | GMB1068 | OR468990 | _ | OR484933 | _ |
| *Xylaria laevis* | HAST 419 | GU324746 | GQ848359 | GQ502695 | GQ455439 |
| *Xylaria leavis* | HAST 95072910 | GU324747 | GQ848360 | GQ502696 | GQ455440 |
| *Xylaria liboensis** | GMB1073 | _ | OR887271 | OR484934 |  |
| *Xylaria liboensis* | GMB0774 | _ | OR887272 | OR484935 |  |
| *Xylaria lindericola* | FCATAS 852 | MZ005635 | MZ031982 | MZ031978 | _ |
| *Xylaria lindericola* | FCATAS 853 | MZ005636 | MZ048749 | MZ031979 | _ |
| *Xylaria liquidambar* | HAST 93090701 | GU300094 | GQ844810 | GQ487702 | GQ421290.1 |
| *Xylaria longissima* | FCATAS 749 | MF774331 | – | – | _ |
| *Xylaria longissima** | IRAN 16582_F | KP218906 | – | – | _ |
| *Xylaria margaretae* | SWUF17-34 2 | MT622779 | _ | MW459237 | MW459217 |
| *Xylaria meliacearum* | JDR_148 | GU300084 | GQ844797 | GQ478219 | _ |
| *Xylaria minima** | SWUF18-3 2 | MT622789 | _ | MW459245 | MW459226 |
| *Xylaria multiplex* | JDR_259 | GU300099 | GQ844815 | GQ487706 | _ |
| *Xylaria muscula* | HAST_520 | GU300087 | GQ844800 | GQ478222 | GQ408909 |
| *Xylaria negundinis** | GMB1082 | OR468993 | OR887273 | OR485583 | _ |
| *Xylaria negundinis* | GMB1166 | OR468992 | OR887274 | OR485584 | _ |
| *Xylaria nigripes* | HAST 653 | GU324755 | GQ853027 | GQ502710 | GQ853045 |
| *Xylaria ochraceostroma* | HAST 401 | EU179869 | GQ853034 | GQ502717 | GQ853052 |
| *Xylaria oligotoma* | HAST 784 | GU300092 | GQ844808 | GQ487700 | GQ421288 |
| *Xylaria ophiopoda* | HAST 93082805 | GU322461 | GQ848344 | GQ495955 | GQ452365 |
| *Xylaria orbiculati** | GMB1083 | OR468995 | OR887275 | OR485585 | _ |
| *Xylaria orbiculati* | GMB1084 | OR468994 | OR887276 | OR485586 | _ |
| *Xylaria ovata** | GMB1085 | OR468998 | OR887277 | _ | _ |
| *Xylaria ovata* | GMB1086 | OR468996 | OR887278 | _ | _ |
| *Xylaria oxyacanthae* | JDR 859 | GU322434 | GQ844820 | GQ495927 | _ |
| *Xylaria oxyacanthae* | L.Z. 2010-502 | HQ414587 | – | – | _ |
| *Xylaria palmicola* | PDD 604 | GU322436 | GQ844822 | GQ495929 | GQ438750 |
| *Xylaria phyllocharis* | HAST 528 | GU322445 | GQ844832 | GQ495938 | GQ449234 |
| *Xylaria plebeja* | HAST 91122401 | GU324740 | GQ848353 | GQ502689 | GQ452374 |
| *Xylaria polymorpha* | JDR 1012 | GU322460 | GQ848343 | GQ495954 | GQ452364 |
| *Xylaria polymorpha* | MM0125909 | FM164944 | _ | – | _ |
| *Xylaria polysporicola** | FCATAS_848 | MZ005592 | MZ031980 | MZ031976 | _ |
| *Xylaria polysporicola* | FCATAS_849 | MZ005591 | MZ031981 | MZ031977 | _ |
| *Xylaria pseudoanisopleura** | GMB1088 | _ | OR887279 | OR485587 |  |
| *Xylaria pseudobambusicola* | GMB1091 | OR469004 | OR887281 | OR485591 | _ |
| *Xylaria pseudobambusicola** | GMB1090 | OR469002 | OR887280 | OR485590 |  |
| *Xylaria pseudocubensis** | GMB1089 | OR468997 | OR887282 | OR485588 | _ |
| *Xylaria pseudocubensis* | GMB0775 | OR468999 | OR887283 | OR485589 | _ |
| *Xylaria pseudoglobosa** | GMB1092 | _ | OR887284 | OR485592 |  |
| *Xylaria pseudohemisphaerica** | GMB1093 | _ | OR887285 | OR485593 |  |
| *Xylaria pseudohypoxylon** | GMB1094 | OR469003 | OR887286 | OR485594 | _ |
| *Xylaria pseudohypoxylon* | GMB0776 | OR469005 | OR887287 | OR485595 | _ |
| *Xylaria puerensis** | GMB1095 | OR469008 | OR887288 | OR485596 | _ |
| *Xylaria puerensis* | GMB1167 | OR469007 | OR887289 | OR485597 | _ |
| *Xylaria qianensis** | GMB1050 | OR469006 | OR753867 | OR484924 | _ |
| *Xylaria qianensis* | GMB1049 | OR469013 | OR753868 | OR484925 | _ |
| *Xylaria qiongzhouensis** | GMB1096 | OR469009 | OR887290 | OR485598 | _ |
| *Xylaria qiongzhouensis* | GMB1097 | _ | OR887291 | OR485599 |  |
| *Xylaria regalis* | HAST 920 | GU324745 | GQ848358 | GQ502694 | GQ452379 |
| *Xylaria reinkingii** | SWUF17-19 1 | MT622769 | _ | MW459234 | _ |
| *Xylaria rhombostroma** | GMB1077 | _ | OR887292 | OR484936 |  |
| *Xylaria rhombostroma* | GMB1078 | _ | OR887293 | OR484937 |  |
| *Xylaria ripicola* | KA11-0060-1 | NR153251 | _ | _ | _ |
| *Xylaria ripicola* | KA11-0060-2 | KM817200 | _ | _ | _ |
| *Xylaria schweinitzii* | HAST 92092023 | GU322463 | GQ848346 | GQ495957 | GQ452367 |
| *Xylaria serratifoliae** | GMB1102 | _ | OR887294 | OR485600 |  |
| *Xylaria shishangensis** | GMB1103 | _ | OR887295 | OR485601 |  |
| *Xylaria shishangensis* | GMB1168 | _ | OR887296 | OR485602 |  |
| *Xylaria shuangjiangensis** | GMB1104 | _ | OR887297 | OR485605 |  |
| *Xylaria shuangjiangensis* | GMB0777 | _ | OR887298 | OR485606 |  |
| *Xylaria shuqunii** | GMB1105 | OR469012 | OR887299 | OR485603 | _ |
| *Xylaria shuqunii* | GMB1106 | OR469011 | OR887300 | OR485604 | _ |
| *Xylaria siamensis* | SWUF17-20 2 | MT622765 | _ | MW459233 | MW459208 |
| *Xylaria sicula f major* | HAST 90071613 | GU300081 | GQ844794 | GQ478216 | GQ408903 |
| *Xylaria sihanonthii** | SWUF18-5 1 | MT622784 | _ | MW459241 | MW459221 |
| *Xylaria sinensis* | GMB1109 | OR469010 | OR887301 | OR485607 | _ |
| *Xylaria sinensis* | GMB0778 | OR469014 | OR887302 | OR485608 | _ |
| *Xylaria* sp | ASMC3 | EU164404 | _ | _ | _ |
| *Xylaria* sp | AAG5 | EU164400 | _ | _ | _ |
| *Xylaria* sp 1 | HAST 642 | GU324759 | GQ853036 | GQ502719 | GQ853054 |
| *Xylaria* sp 3 | HAST 722 | GU324756 | GQ853029 | GQ502712 | GQ853047 |
| *Xylaria* sp 4 | HAST 646 | GU324757 | _ | GQ502715 | GQ853050 |
| *Xylaria* sp 5 | HAST 650 | GU324758 | GQ853033 | GQ502716 | GQ853051 |
| *Xylaria* sp 6 | 258 JDR | GU300082 | GQ844795 | GQ478217 | GQ408904 |
| *Xylaria striata* | HAST 304 | GU300089 | GQ844803 | GQ478224 | GQ421284 |
| *Xylaria subescharoidea* | HAST 660 | GU324754 | GQ853025 | GQ502708 | GQ853043 |
| *Xylaria subescharoidea* | HAST 99060401-1188 | MN655980 | MN656982 | MN656984 | MN656986 |
| *Xylaria subintraflava* | SWUF16-4 3 | MT622762 | _ | MW459230 | MW459204 |
| *Xylaria subintraflava* | SWUF17-22 2 | MT622764 | _ | MW459231 | MW459206 |
| *Xylaria tentaculata* | KA12-0530 | KM077162 | _ | – | _ |
| *Xylaria tentaculata* | KA13-1324 | KM077163 | _ | – | _ |
| *Xylaria tentaculata* | KA13-1325 | KM077164 | _ | – | _ |
| *Xylaria terricola* | YMJ 1375 | MF577042 | MF577043 | MF577044 | MF577045 |
| *Xylaria thienhirunae** | SWUF16-6 2 | MT622770 | _ | MW459235 | MW459210 |
| *Xylaria thienhirunae* | SWUF16-7 2 | MT622772 | _ | _ | _ |
| *Xylaria tongrenensis** | GMB1114 | OR469015 | OR887303 | OR485609 | _ |
| *Xylaria tongrenensis* | GMB1169 | OR469016 | OR887304 | OR485610 | _ |
| *Xylaria umbellate** | GMB1116 | OR469019 | OR887305 | OR485611 | _ |
| *Xylaria umbellata* | GMB1170 | OR469020 | OR887306 | OR485612 | _ |
| *Xylaria venosula* | HAST 94080508 | EF026149 | GQ844806 | EF025617 | EF025602 |
| *Xylaria venustula* | HAST 88113002 | GU300091 | GQ844807 | GQ487699 | GQ421287 |
| *Xylaria vinacea** | SWUF18-2.1 | MT622781 | _ | MW459239 | MW459219 |
| *Xylaria vinacea* | SWUF18-2 10 | MT622783 | _ | MW459240 | MW459220 |
| *Xylaria xishuiensis** | GMB1120 | OR469021 | _ | OR485613 | _ |
| *Xylaria xishuiensis* | GMB0779 | OR469023 | _ | OR485614 | _ |
| *Xylaria xylarioides* | GUM_1151 | KP218909 | _ | – | _ |
| *Xylaria yaorenshanensis** | GMB1125 | _ | OR887307 | OR485615 |  |
| *Xylaria yinggelingensis** | GMB1126 | _ | OR887308 | OR485616 |  |
| *Xylaria yinggelingensis* | GMB1127 | _ | OR887309 | OR485617 |  |
| *Xylaria yumingii** | GMB1128 | OR469022 | _ | OR485618 | _ |
| *Xylaria yunnanensis** | GMB1129 | OR469026 | OR887310 | OR485619 | _ |
| *Xylaria yunnanensis* | GMB0780 | OR469025 | OR887311 | OR485620 | _ |
| *Xylaria zangmui** | GMB1130 | OR469024 | OR753880 | OR485621 | _ |
| *Xylaria zangmui* | GMB0781 | OR469028 | OR753881 | OR485622 | _ |
| *Xylaria zonghuangii** | GMB1131 | OR469030 | OR753878 | OR485623 | _ |
| *Xylaria zonghuangii* | GMB1132 | OR469027 | OR753879 | OR485624 | _ |

**Table S2.** List of taxa used for the phylogenetic reconstruction (Hypoxylaceae). GenBank accession numbers, specimen number, origin, status and reference. Newly generated sequences are in **bold,** and the type strains are marked with*. “_” Sequences were not available.

| **Taxon** | **Specimen/culture** | **ITS** | **LSU** | ***rpb2*** | ***tub*** |
| --- | --- | --- | --- | --- | --- |
| *Annulohypoxylon albidiscum* | MFLUCC 15-0645 | KU852741 | _ | _ | _ |
| *Annulohypoxylon annulatum** | CBS 140775 | KU604559 | KY610418 | KY624263 | KU159523 |
| *Annulohypoxylon annulatum* | DSM 103479 | KX376330 | MK287546 | MK287559 | KX376353 |
| *Annulohypoxylon areolatum** | MFLUCC 14-1233 | NR_153554 | _ | _ | KX376344 |
| *Annulohypoxylon atroroseum* | MFLUCC 14-1220 | KP401581 | _ | _ | KP401588 |
| *Annulohypoxylon bahnphadengense* | STMA 13115 | KX376338 | _ | _ | KX376347 |
| *Annulohypoxylon elevatidiscus** | YMJ 90080706 | _ | _ | _ | AY951656 |
| *Annulohypoxylon fulvum* | MUCL 54617 | KX376336 | _ | _ | KX376355 |
| *Annulohypoxylon fulvum** | MUCL 54622 | KX376337 | _ | _ | KX376354 |
| *Annulohypoxylon leptascum* | MFLUCC 13-587 | KU604576 | _ | _ | KU604580 |
| *Annulohypoxylon macrosporum* | ST2584 | DQ322097 | _ | _ | _ |
| *Annulohypoxylon maeteangense* | CBS 123835 | KX376322 | _ | _ | _ |
| *Annulohypoxylon massivum** | MUCL 47218 | AM749938 | _ | _ | KC977276 |
| *Annulohypoxylon michelianum* | CBS 119993 | KX376320 | KY610423 | KY624234 | KX271239 |
| *Annulohypoxylon michelianum* | DSM 103483 | KX376339 | _ | _ | KX376346 |
| *Annulohypoxylon microdiscum* | BCRC 34018 | EF026137 | _ | _ | AY951660 |
| *Annulohypoxylon moriforme* | CBS 123579 | KX376321 | KY610425 | KU684279 | KX271261 |
| *Annulohypoxylon moriforme* | STMA 14065 | KU604561 | _ | _ | KU159525 |
| *Annulohypoxylon nitens* | AXL030 | KJ934991 | KJ934992 | KJ934994 | KJ934993 |
| *Annulohypoxylon nitens* | BCRC 34021 | EF026138 | _ | _ | AY951663 |
| *Annulohypoxylon nouraguense** | MUCL 54607 | KX376335 | _ | _ | KX376348 |
| *Annulohypoxylon nouraguense* | MUCL 54608 | KX376334 | _ | _ | _ |
| *Annulohypoxylon palmicola* | MFLUCC 11-0020 | KT369002 | KT369003 | _ | _ |
| *Annulohypoxylon purpureonitens* | ST2448 | DQ223756 | _ | _ | _ |
| *Annulohypoxylon purpureonitens* | ST2485 | DQ223757 | _ | _ | _ |
| *Annulohypoxylon squamulosum** | BCRC 34022 | EF026139 | _ | _ | AY951665 |
| *Annulohypoxylon stygium* | MFLUCC 12-0826 | KJ940870 | KJ940869 | KJ940868 | KJ940867 |
| *Annulohypoxylon stygium* | MUCL 54600 | KC968940 | KY610474 | KY624291 | KC977304 |
| *Annulohypoxylon subnitens* | MUCL 54594 | KX376333 | _ | _ | _ |
| *Annulohypoxylon substygium** | MUCL 51708 | KC968915 | _ | _ | KC977285 |
| *Annulohypoxylon substygium* | STMA 14066 | KU604575 | KY610426 | _ | KU159526 |
| *Annulohypoxylon thailandicum** | MFLUCC 13-0118 | KP744434 | KP744476 | _ | KX376349 |
| ***Annulohypoxylon thailandicum*** | **MFLUCC 24-0086** | **PP824657** | **_** | **PP838802** | **_** |
| *Annulohypoxylon truncatum* | CBS 140777 | KU604560 | _ | _ | KU159524 |
| *Annulohypoxylon truncatum* | DSM 107925 | MK287531 | MK287543 | MK287556 | MK287569 |
| *Annulohypoxylon violaceopigmentum** | MFLUCC 14-1225 | KX376326 | _ | _ | KX376343 |
| *Annulohypoxylon viridistratum** | MFLUCC 14-1224 | KX376325 | _ | _ | KX376342 |
| *Annulohypoxylon yungensis** | STMA 14046 | KX376323 | _ | _ | KX376340 |
| *Daldinia concentrica* | ATCC 36659 | AF201708 | U47828 | DQ368651 | DQ368623 |
| *Daldinia eschscholtzii** | MFLUCC 19-0154 | MK587660 | MK587747 | MK625011 | MK636690 |
| *Daldinia eschscholtzii* | MUCL 45435 | JX658484 | KY610437 | KY624246 | KC977266 |
| *Daldinia placentiformis* | MUCL 47603 | AM749921 | KY610440 | KY624249 | KC977278 |
| *Entonaema liquescens* | ATCC 46302 | KY610389 | KY610443 | KY624253 | KX271248 |
| *Hypomontagnella monticulosa* | CLL 205 | MK131719 | MK131717 | MK135890 | MK135892 |
| *Hypomontagnella monticulosa** | MUCL 54604 | KY610404 | KY610487 | KY624305 | KX271273 |
| *Hypomontagnella submonticulosa* | CBS 115280 | KC968923 | KY610457 | KY624226 | KC977267 |
| *Hypoxylon addis** | MUCL 52797 | KC968931 | _ | _ | KC977287 |
| *Hypoxylon aurantium* | MFLU 16-1202 | NR_166287 | MN017878 | _ | _ |
| *Hypoxylon aurantium* | MFLU 18-531 | MN047115 | MN017879 | _ | MN077081 |
| *Hypoxylon baruense* | MC66 | MN056428 | _ | _ | MK908142 |
| *Hypoxylon begae* | BCRC 34051 | JN660820 | _ | _ | AY951704 |
| *Hypoxylon begae* | S99 | KT224877 | _ | _ | _ |
| *Hypoxylon bellicolor* | MC36 | MN056425 | _ | _ | MK908139 |
| *Hypoxylon blackburniae* | BRIP 72467b | NR_182618 | OP602214 | _ | _ |
| *Hypoxylon brevisporum* | BCRC 33809 | JN660821 | _ | _ | AY951705 |
| *Hypoxylon calileguense* | STMA 14059 | KU604566 | _ | _ | KU604579 |
| *Hypoxylon calileguense* | STMA 14070 | KU604565 | _ | _ | KU604578 |
| *Hypoxylon carneum* | MUCL 54177 | KY610400 | KY610480 | KY624297 | KX271270 |
| *Hypoxylon carneum* | YMJ 30 | JN660822 | _ | _ | AY951706 |
| *Hypoxylon cercidicola* | CBS 119009 | KC968908 | KY610444 | KY624254 | KC977263 |
| *Hypoxylon cinnabarinum* | BCRC 34055 | JN979409 |  | _ | AY951708 |
| *Hypoxylon cinnabarinum* | MC97 | MN056429 | _ | _ | MK908143 |
| *Hypoxylon crocopeplum* | BCRC 34056 | JN979410 | _ | _ | AY951710 |
| *H. damuense* | FCATAS 4207 | ON075427 | ON075433 | ON093251 | ON093245 |
| *H. damuense* | FCATAS 4320 | ON075428 | ON075434 | ON093252 | ON093246 |
| *Hypoxylon dieckmannii* | YMJ 45 | JN979412 | _ | _ | AY951712 |
| *Hypoxylon dieckmannii* | YMJ 89041203 | JN979413 | _ | _ | AY951713 |
| *Hypoxylon erythrostroma* | MUCL 53759 | KC968910 | _ | _ | KC977296 |
| *Hypoxylon erythrostroma* | YMJ 90080602 | JN979416 | _ | _ | AY951716 |
| *Hypoxylon fendleri* | DSM 107923 | _ | _ | MK287554 | MK287567 |
| *Hypoxylon fendleri* | DSM 107927 | MK287533 | MK287545 | MK287558 | MK287571 |
| *Hypoxylon fendleri* | MUCL 54792 | KF234421 | KY610481 | KY624298 | KF300547 |
| *Hypoxylon ferrugineum* | CBS 141259 | KX090079 | _ | _ | KX090080 |
| *Hypoxylon flavoargillaceum* | STMA 14062 | KU604577 | _ | _ | KU159532 |
| *Hypoxylon fragiforme** | MUCL 51264 | KC477229 | KM186295 | MK887342 | KX271282 |
| *Hypoxylon fraxinophilum** | MUCL 54176 | KC968938 | _ | _ | KC977301 |
| *Hypoxylon fulvo-sulphureum** | MFLUCC 13-0589 | KP401576 | _ | _ | KP401584 |
| *Hypoxylon fuscopurpureum* | BCRC 34067 | JN979421 | _ | _ | AY951721 |
| *Hypoxylon fuscum** | STMA 13090 | KY610401 | KY610482 | KY624299 | KX271271 |
| *Hypoxylon fuscum* | YMJ 23 | JN979424 | _ | _ | AY951724 |
| *Hypoxylon griseobrunneum* | CBS 117742 | KC968917 | KY610446 | KY624256 | KC977271 |
| *Hypoxylon griseobrunneum** | CBS 331.73 | KY610402 | KY610483 | KY624300 | KC977303 |
| *Hypoxylon griseobrunneum* | BCRC 34050 | JN660819 | _ | _ | AY951703 |
| *Hypoxylon griseobrunneum* | Smeg4 | MF663791 | _ | _ | _ |
| *Hypoxylon guilanense** | 989 | MT214997 | MT214992 | MT212235 | MT212239 |
| *Hypoxylon haematostroma* | MUCL 47600 | AM749924 | KY610447 | KY624257 | KC977279 |
| *Hypoxylon haematostroma** | MUCL 53301 | KC968911 | KY610484 | KY624301 | KC977291 |
| *Hypoxylon hinnuleum* | DSM 107926 | MK287532 | MK287544 | MK287557 | MK287570 |
| *Hypoxylon hinnuleum* | DSM 107932 | MK287535 | MK287547 | MK287560 | MK287573 |
| *Hypoxylon hongheense ** | KUMCC 21-0452 | OM001333 | OM001334 | ON39008 | ON468655 |
| *Hypoxylon hongheense* | KUN-HKAS 122663 | OM001336 | OM001339 | ON392009 | ON468656 |
| *Hypoxylon howeanum* | MC62 | MN056427 | _ | _ | MK908144 |
| *Hypoxylon howeanum* | MUCL 47599 | AM749928 | KY610448 | KY624258 | KC977277 |
| ***Hypoxylon hypomiltum*** | **MFLUCC 24-0088** | **PP824656** | **_** | **_** | **PP831684** |
| *Hypoxylon hypomiltum* | MUCL 51845 | KY610403 | KY610449 | KY624302 | KX271249 |
| *Hypoxylon inaequale** | HKAS 123207 | NR_185719 | _ | _ | OQ652093 |
| *Hypoxylon investiens* | CBS 118183 | KC968925 | KY610450 | KY624259 | KC977270 |
| *Hypoxylon investiens* | CBS 118185 | KC968924 | KY610451 | KY624260 | KC977269 |
| *Hypoxylon jaklitschii** | CBS 138916 | KM610290 | _ | _ | KM610304 |
| *Hypoxylon jianfengense** | FCATAS845 | MW984546 | MZ029707 | MZ047260 | MZ047264 |
| *Hypoxylon jecorinum* | YMJ 39 | JN979429 | _ | _ | AY951731 |
| *Hypoxylon lateripigmentum** | MUCL 53304 | KC968933 | KY610486 | _ | KC977290 |
| *Hypoxylon lateripigmentum* | CBS 129031 | _ | _ | KY624304 | _ |
| *Hypoxylon lenormandii* | CBS 119003 | KC968943 | KY610452 | KY624261 | KC977273 |
| *Hypoxylon lenormandii* | CBS 135869 | KM610281 | KY610453 | KY624262 | KM610295 |
| *Hypoxylon liviae** | CBS 115282 | KC968922 | _ | _ | KC977265 |
| *Hypoxylon lividicolor** | BCRC 34076 | JN979432 | _ | _ | AY951734 |
| *Hypoxylon macrosporum* | YMJ 47 | JN979434 | _ | _ | AY951736 |
| *Hypoxylon mangrovei** | MFLU 18-0559 | NR_166288 | NG_068299 | _ | MN077053 |
| *Hypoxylon mangrovei* | MFLU 18-0575 | MN047117 | MN017881 | _ | MN077054 |
| *Hypoxylon medogense* | FCATAS 4320 | ON075426 | ON075432 | ON093250 | ON093244 |
| *Hypoxylon medogense** | FCATAS 4061 | ON075425 | ON075431 | ON093249 | ON093243 |
| *Hypoxylon munkii* | MUCL 53315 | KC968912 | _ | _ | KC977294 |
| *Hypoxylon munkii* | YMJ 90080403 | JN979436 | _ | _ | AY951738 |
| *Hypoxylon musceum* | MUCL 53765 | KC968926 | _ | KY624306 | KC977280 |
| *Hypoxylon ochraceum** | MUCL 54625 | KC968937 | _ | − | KC977300 |
| *Hypoxylon olivaceopigmentum** | DSM 107924 | MK287530 | MK287542 | MK287555 | MK287568 |
| *Hypoxylon papillatum* | ATCC 58729 | KC968919 | KY610454 | KY624223 | KC977258 |
| *Hypoxylon perforatum* | CBS 115281 | KY610391 | KY610455 | KY624224 | KX271250 |
| *Hypoxylon perforatum* | DSM 107930 | MK287529 | MK287540 | MK287553 | MK287566 |
| *Hypoxylon petriniae** | CBS 114746 | KY610405 | KY610491 | KY624279 | KX271274 |
| *Hypoxylon petriniae* | YMJ 356 | JQ009309 | _ | _ | AY951743 |
| *Hypoxylon pilgerianum* | STMA 13455 | KY610412 | _ | KY624308 | KY624315 |
| *Hypoxylon pilgerianum* | YMJ 92042505 | JQ009310 | _ | _ | AY951744 |
| *Hypoxylon polyporoideum* | YMJ 15 | JQ009311 | _ | _ | AY951747 |
| *Hypoxylon polyporoideum* | YMJ 56 | JQ009312 | _ | _ | AY951748 |
| *Hypoxylon porphyreum* | CBS 119022 | KC968921 | KY610456 | KY624225 | KC977264 |
| *Hypoxylon pulicicidum** | CBS 122622 | JX183075 | KY610492 | KY624280 | JX183072 |
| *Hypoxylon rickii** | MUCL 53309 | KC968932 | KY610416 | KY624281 | KC977288 |
| *Hypoxylon rickii* | YMJ 25 | JQ009313 | _ | _ | AY951750 |
| *Hypoxylon rubiginosum** | MUCL 52887 | KC477232 | KY610469 | KY624266 | KY624311 |
| *Hypoxylon rubiginosum* | YMJ 24 | EF026143 | _ | JX507791 | AY951751 |
| *Hypoxylon samuelsii** | MUCL 51843 | KC968916 | KY610466 | KY624269 | KC977286 |
| *Hypoxylon spegazzianum** | STMA 14082 | KU604573 | _ | _ | KU604582 |
| *Hypoxylon sporistriatatunicum** | MC50 | MN056426 | _ | _ | MK908140 |
| *Hypoxylon subgilvum* | YMJ 246 | JQ009314 | _ | _ | AY951754 |
| *Hypoxylon subrutiloides* | F 202416 | FJ185304 | _ | _ | FJ185281 |
| *Hypoxylon subticinense* | MUCL 53752 | KC968913 | _ | _ | KC977297 |
| *Hypoxylon szostakii* | BRIP 72527b | NR_182620 | OP598063 | _ | _ |
| *Hypoxylon texense* | DSM 107928 | MK287527 | MK287538 | MK287551 | MK287564 |
| *Hypoxylon texense** | DSM 107933 | MK287536 | MK287548 | MK287561 | MK287574 |
| *Hypoxylon ticinense* | MUCL 47714 | KY610410 | KY610470 | KY624267 | KX271259 |
| *Hypoxylon ticinense* | YMJ 313 | JQ009317 | KY610471 | _ | AY951757 |
| *Hypoxylon trugodes** | MUCL 54794 | KF234422 | KY610493 | KY624282 | KF300548 |
| *Hypoxylon trugodes* | YMJ 57 | JQ009319 | _ | _ | AY951759 |
| *Hypoxylon ulmophilum* | BCRC 34100 | JQ009320 | _ | _ | AY951760 |
| *Hypoxylon vogesiacum* | CBS 115273 | KC968920 | _ | KY624283 | KX271275 |
| *Hypoxylon wujianggensis** | GMBC0213 | MT568854 | MT568853 | MT585802 | MT572481 |
| *Hypoxylon wujianggensis* | GMBC0214 | MT568856 | MT568855 | MT585803 | MT585801 |
| *Hypoxylon zangii* | FCATAS 4319 | ON075424 | ON075430 | ON093248 | ON093242 |
| *Hypoxylon zangii** | FCATAS 4029 | ON075423 | ON075429 | ON093247 | ON093241 |
| *Hypoxylon zhaotongensis** | GMBCC1168 | OP597690 | OP598100 | OP615662 | OP615660 |
| *Jackrogersella cohaerens* | YMJ 310 | EF026140 | _ | GQ844766 | AY951655 |
| *Jackrogersella minutella* | CBS_119015 | JX658447 | KY610424 | KY624235 | KX271240 |
| *Jackrogersella multiformis** | CBS 119016 | KC477234 | KY610473 | KY624290 | KX271262 |
| *Pyrenopolyporus hunteri** | MUCL 52673 | KY610421 | KY610472 | KY624309 | KU159530 |
| *Pyrenopolyporus laminosus** | MUCL 53305 | KC968934 | KY610485 | KY624303 | KC977292 |
| *Pyrenopolyporus laminosus* | TBRC 8871 | MH938527 | MH938536 | MK165424 | MK165415 |
| *Rhopalostroma angolense* | CBS 126414 | KY610420 | KY610459 | KY624228 | KX271277 |
| *Rhopalostroma angolense* | MUCL52664 | FN821965 | KM186298 | KM186297 | KM186299 |
| *Rostrohypoxylon terebratum** | CBS 119137 | DQ631943 | _ | DQ631954 | DQ840097 |
| *Ruwenzoria pseudoannulata** | MUCL 51394 | KY610406 | KY610494 | KY624286 | KX271278 |
| *Thamnomyces dendroidea** | CBS 123578 | FN428831 | KY610467 | KY624232 | KY624313 |
| *Xylaria hypoxylon* | 95082001 | GU300095 | _ | GQ844811 | GQ487703 |
| *Xylaria hypoxylon** | CBS 122620 | KY610407 | KY610495 | KY624231 | KX271279 |

**Table S3.** List of taxa used for the phylogenetic reconstruction (Oxydothidaceae). GenBank accession numbers, specimen number, origin, status and reference. Newly generated sequences are in **bold,** and the type strains are marked with*. “_” Sequences were not available.

| Taxon | Specimen/culture | ITS | LSU | SSU |
| --- | --- | --- | --- | --- |
| *Oxydothis calamicola* | MFLUCC 14-1165 | _ | KY206761 | KY206767 |
| *Oxydothis chinensis* | ZHKUCC 22-0134 | OR164912 | OR164957 | _ |
| *Oxydothis cyrtostachicola* | MRC 0007 | DQ660334 | DQ660337 | _ |
| *Oxydothis daemonoropsicola* | MRC 0005 | DQ660335 | DQ660338 | _ |
| *Oxydothis fortunei* | GMB0389 | ON510944 | ON510945 | _ |
| *Oxydothis fortunei* | GMB0315 | ON479893 | ON479894 | _ |
| *Oxydothis frondicola* | HKUCC 1001 | AF009803 | AY083835 | AY083818 |
| *Oxydothis garethjonesii* | MFLUCC 15-0287 | KY206773 | KY206762 | KY206768 |
| *Oxydothis hoehnelii* | HKUCC 3854 | _ | DQ810226 | DQ810259 |
| *Oxydothis inaequalis* | MRC 0004 | DQ660336 | DQ660339 | _ |
| *Oxydothis metroxylonicola* | MFLUCC 15-0281 | KY206774 | KY206763 | KY206769 |
| *Oxydothis metroxylonis* | MFLUCC 15-0283 | KY206775 | KY206764 | KY206770 |
| *Oxydothis narathiwatensis* | **MFLUCC 24-0085** | **PP824654** | **PP824658** | **PP824659** |
| *Oxydothis palmicola* | MFLUCC 15-0806 | KY206776 | KY206765 | KY206771 |
| *Oxydothis phoenicis* | MFLUCC 18-0269 | MK088065 | MK088061 | MK088063 |
| *Oxydothis phoenicis* | MFLUCC 18-0270 | MK088066 | MK088062 | MK088064 |
| *Oxydothis rhapidicola* | MFLUCC 14-0616 | _ | KY206766 | KY206772 |
| *Oxydothis* sp*.* | IFO 32218 | _ | DQ810225 | DQ810261 |
| *Oxydothis yunnanensis* | GZUCC 0127 | ON176681 | ON176684 | _ |
| *Vialaea mangiferae* | MFLUCC 12-0808 | NR_171903 | NG_073594 | _ |
| *Vialaea minutella* | BRIP 56959 | KC181926 | KC181924 | _ |
